# Global, regional, and national burden inequality of chronic kidney disease, 1990–2021: a systematic analysis for the global burden of disease study 2021

**DOI:** 10.3389/fmed.2024.1501175

**Published:** 2025-01-15

**Authors:** Jingxun Guo, Zhen Liu, Pengjun Wang, Heming Wu, Kai Fan, Jianbo Jin, Lan Zheng, Zeyu Liu, Renyi Xie, Cheng Li

**Affiliations:** ^1^Eye Institute and Affiliated Xiamen Eye Center, School of Medicine, Xiamen University, Xiamen, Fujian, China; ^2^Fujian Provincial Key Laboratory of Ophthalmology and Visual Science and Ocular Surface and Corneal Diseases, Xiamen, Fujian, China; ^3^State Key Laboratory of Molecular Vaccinology and Molecular Diagnostics, School of Public Health, Xiamen University, Xiamen, China; ^4^Department of Basic Medical Sciences, School of Medicine, Xiamen University, Xiamen, China; ^5^Xiamen Clinical Research Center for Eye Diseases, Xiamen, Fujian, China; ^6^Translational Medicine Institute of Xiamen Eye Center of Xiamen University, Xiamen, Fujian, China; ^7^Huaxia Eye Hospital of Quanzhou, Quanzhou, Fujian, China

**Keywords:** chronic kidney disease, global burden of disease, prevalence, incidence, mortality, disability-adjusted life years

## Abstract

**Background:**

Chronic kidney disease (CKD) is a significant global health issue, often linked to diabetes, hypertension, and glomerulonephritis. However, aggregated statistics can obscure heterogeneity across subtypes, age, gender, and regions. This study aimed to analyze global CKD trends from 1990 to 2021, focusing on age, gender, socio-demographic index (SDI), and regional variations.

**Methods:**

Data were extracted from the Global Burden of Disease (GBD) 2021 database, covering prevalence, incidence, mortality, and disability-adjusted life years (DALYs). These were presented as counts per 100,000 population and age-standardized rates, with uncertainty intervals (UIs) to highlight variability. Joinpoint regression was used to assess trends over the 30-year period.

**Results:**

In 2021, global CKD prevalence was 359 million, with 11.13 million new cases, 1.53 million deaths, and 44.45 million DALYs—up 92, 156, 176, and 114% since 1990. While prevalence slightly declined, incidence, mortality, and DALYs increased significantly. CKD burden varied by region and age, with notable gender disparities.

**Conclusion:**

The study highlights a dramatic rise in CKD burden linked to population growth and aging, emphasizing the need for targeted treatment and effective global healthcare policies.

## Introduction

Chronic kidney disease (CKD) is characterized by abnormalities in kidney structure or function lasting more than 3 months, with significant health implications (1). CKD can result from a variety of factors, including clinical, sociodemographic, and environmental risks. Common causes include diabetes, hypertension, and glomerulonephritis, as identified in previous studies (2). Its high incidence, prevalence, and mortality rates have made CKD a pressing global public health challenge. Persistent disease burden stems from low awareness, insufficient prevention efforts, and limited treatment access. Moreover, CKD is a major contributor to reduced quality of life, alongside conditions such as cardiovascular diseases, cancer, and diabetes (3).

Recent studies, such as GBD 2019, have expanded our understanding of CKD by analyzing its subgroups (2). However, existing research often falls short in regional representation, longitudinal analyses, and the identification of key inflection points in disease trends—areas critical for effective policy development.

To address these gaps, this study utilizes GBD 2021 data, which offers a broader time frame (from 1990 to 2021) and more comprehensive coverage of CKD subtypes and etiologies. Compared to GBD 2019, the updated dataset provides refined estimates and expanded granularity, enabling deeper insights into global and regional CKD burden trends. To enhance the analysis, this study employs detailed subgroup stratifications by gender, age, and socio-demographic index (SDI). Additionally, Joinpoint regression analysis is applied to identify critical shifts in CKD burden trends over time. These methodological advancements provide a robust foundation for tailored prevention and management strategies.

This study aims to analyze the current status and trends in the burden of CKD and its subtypes from global, regional, and national perspectives, with the goal of increasing understanding of the health impact of CKD worldwide and providing a basis for formulating targeted prevention and treatment strategies.

## Materials and methods

### Data source and disease definition

**Figure 1:**
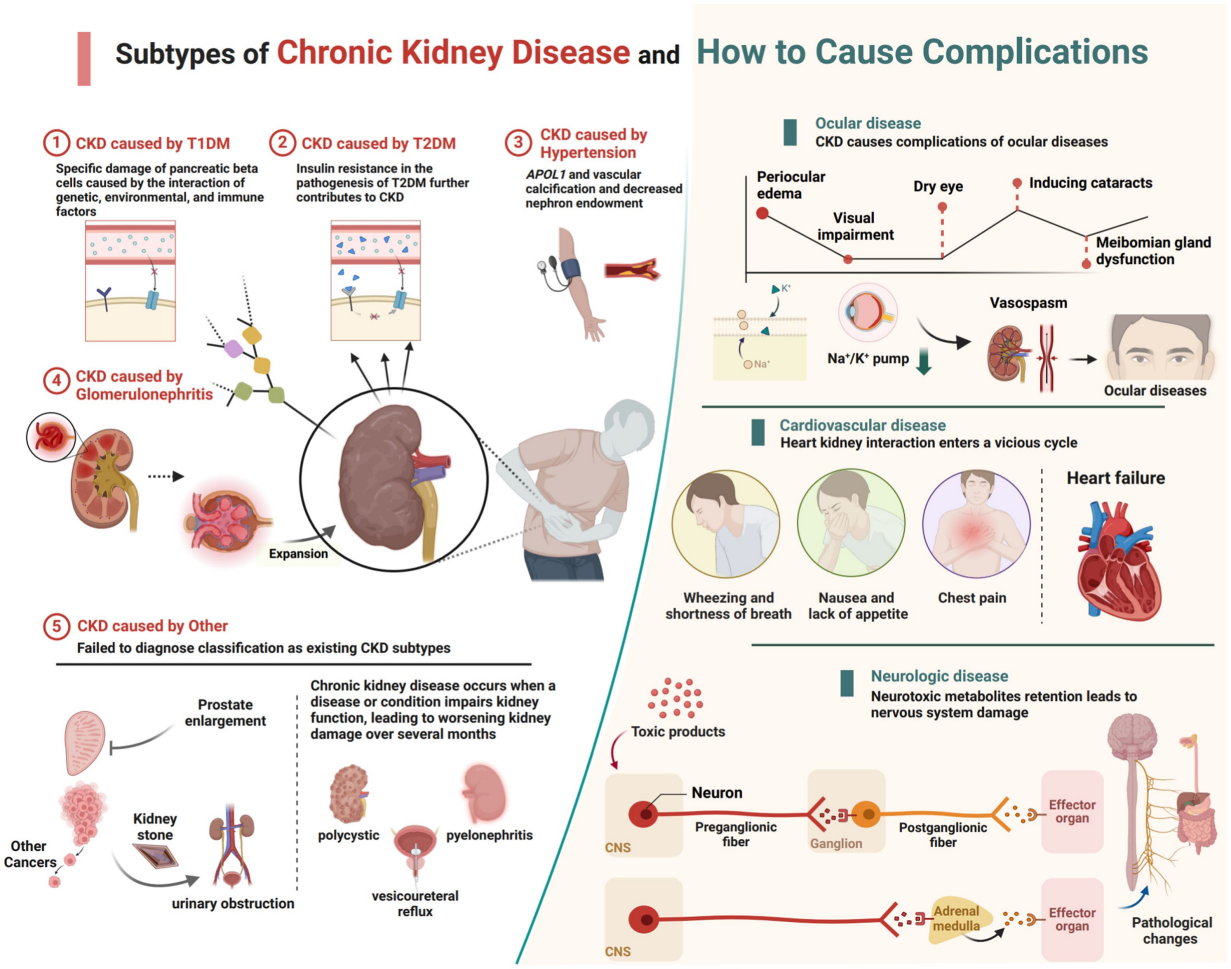
The current subtypes of CKD featured in GBD 2021 and the complications that lead to other systemic diseases (created with BioRender.com).

The CKD data analyzed in this study were sourced from GBD 2021, which provides the latest epidemiological estimates of health hazards for 371 diseases, injuries, and impairments as well as 88 risk factors across 204 countries and regions from 1990 to 2021 (4). All data were freely accessible via the Global Health Data Exchange (GHDx).^1^ Compared to GBD 2019, GBD 2021 re-extracted data from the European Renal Association–European Dialysis and Transplant Association for 1998–2017 using global sex-specific coefficients, finer staging of dialysis, and narrower age ranges to enhance the accuracy of the database estimation models. GBD 2021 categorizes causes into four levels, ranging from Level 1 communicable, maternal, neonatal, and nutritional diseases to Level 4 latent tuberculosis infection (4). In GBD 2021, CKD is classified as a Level 3 cause, with five subtypes causing CKD burden categorized as Level 4 causes: type 1 diabetes, type 2 diabetes, glomerulonephritis, hypertension, and other causes (Figure 1). These specific causes were selected based on extensive epidemiological evidence indicating that diabetes, glomerulonephritis, and hypertension are major contributors to CKD globally, and are closely associated with its onset and progression. According to GBD 2021, CKD is defined as a condition with an estimated glomerular filtration rate below 60 mL/min/1.73 m^2^ or an albumin to creatinine ratio greater than 30 mg/g, encompassing disability and death, as well as those undergoing dialysis or kidney transplantation for end-stage renal disease.^2^ This study incorporated CKD and its five subtypes, aligning with the International Statistical Classification of Diseases and Related Health Problems, 10th Revision (ICD-10) definition.

### Socio-demographic index

This study also utilizes the Sociodemographic Index (SDI), a quantitative measure of social population development in a region based on income, education, and fertility rates (5). GHDx categorizes 204 countries and regions into five groups based on their SDI values: low SDI (<0.45), low-middle SDI (≥0.45 and <0.61), middle SDI (≥0.61 and <0.69), high-middle SDI (≥0.69 and <0.80), and high SDI (≥0.80) using a scale from 0 to 1. A higher SDI value indicates a higher level of development for a country or region (6).

### Joinpoint regression analysis

In this study, Joinpoint software (version 5.2.0) was employed to conduct Joinpoint regression analysis on the temporal trends of age-standardized rates of global CKD and its subtypes from 1990 to 2021. This analysis aimed to assess the global trends in the prevalence, incidence, mortality rates, and Disability-Adjusted Life Years (DALYs) associated with CKD The study calculated the Annual Percent Change (APC) and Average Annual Percent Change (AAPC) with their corresponding 95% confidence intervals (CI) for the entire study period (1990–2021) and different segmented periods. From a statistical perspective, APC or AAPC estimates with a lower bound of the 95% CI exceeding zero indicate an increasing trend within the specified interval. Conversely, a decreasing trend is observed when the upper bound of the 95% CI of the APC or AAPC estimates falls below zero. When the 95% CI of APC or AAPC was zero, a stable trend was suggested. The significance level for testing was set at *p* < 0.05 (7, 8).

### Study variables and statistical analysis

Previous studies have explored the burden of diseases associated with CKD and provided valuable insights into this field (2, 9). In our latest research, we conducted a comprehensive assessment to quantify the global burden of CKD, including case numbers, prevalence, incidence, mortality rates, Disability-Adjusted Life Years (DALYs), age-standardized rates (per 100,000 population), Estimated Annual Percentage Change (EAPC), Average Annual Percentage Changes (AAPCs), and their respective 95% uncertainty intervals (UI) (10). Furthermore, this study delved into the demographic variables of CKD across 21 regions, categorized into five SDI regions, and encompassing 204 countries and territories. CKD types and subtypes were analyzed across different age groups and genders. Differences were considered statistically significant at *p* < 0.05 (two-tailed).

This study utilized R software (version 4.4.0) for data cleaning, computation, and visualization using the ggplot2 package. Trend graphs were plotted using GraphPad Prism (version 8.0.2), and the final editing was performed using Adobe Illustrator software (version CC2021).

Since data from the GBD database are publicly available, this study did not require informed consent from patients or institutions.

## Results

### Global level

In 2021, the global number of cases of CKD was 673,722,703 (95% UI: 629,095,119–722,364,096), with an age-standardized prevalence rate (ASPR) of 8,006 per 100,000 population (95% UI: 7,482,12–8,575,62) (Table 1). The global incidence of CKD was 19,935,038 per 100,000 population (95% UI: 18,702,793–21,170,794), with an age-standardized incidence rate (ASIR) of 233.56 per 100,000 population (95% UI: 220.02–247.24) (Table 2). CKD-related death totaled 1,527,639 per 100,000 population worldwide (95% UI: 1,389,377–1,638,914), corresponding to an age-standardized mortality rate (ASMR) of 18.5 per 100,000 population (95% UI: 16.72–19.85) (Table 3). Additionally, the number of CKD DALYs worldwide was 44,453,684 per 100,000 population (95% UI: 40,840,762–48,508,462), with an age-standardized DALY rate (ASDR) of 529.62 per 100,000 population (95% UI, 486.25–577.42) (Table 4).

**Table 1:** Global CKD prevalence by SDI and regions in 1990 and 2021, as well as changes in trends from 1990 to 2021.

	1990		2021		1990–2021 EAPC, No. (95%CI)
	Prevalence cases (95%UI)	ASPR (Per 100,000 population)	Prevalence cases (95%UI)	ASPR (Per 100,000 population)	
Sex					
Female	188,185,026 (175,770,189–201,580,675)	8,293.08 (7,778.75–8,870.27)	358,777,424 (335,684,717–383,643,812)	8,182.65 (7,653.14–8,764.76)	0 (−0.04 to 0.03)
Male	162,777,648 (151,189,911–174,916,268)	7,812.80 (7,301.88–8,356.28)	314,945,280 (293,385,891–338,118,606)	7,808.96 (7,288.71–8,366.6)	0.03 (0–0.05)
Global	350,962,674 (326,973,785–376,155,723)	8,072.75 (7,560.37–8,634.07)	673,722,703 (629,095,119–722,364,096)	8,006 (7,482.12–8,575.62)	0.01 (−0.02 to 0.04)
Region					
Africa					
Central Sub-Saharan Africa	2,633,228 (2,443,845–2,831,336)	9,221.98 (8,598.75–9,806.74)	6,811,951 (6,363,747–7,313,187)	9,165.2 (8,640.85–9,709.73)	−0.08 (−0.09 to −0.06)
Eastern Sub-Saharan Africa	5,981,606 (5,488,507–6,503,182)	5,677.22 (5,266.15–6,113.13)	15,017,846 (13,741,759–16,422,384)	5,821.27 (5,404.49–6,293.6)	0.11 (0.1–0.12)
Western Sub-Saharan Africa	9,003,658 (8,381,320–9,663,357)	8,310.65 (7,767.39–8,851.61)	22,472,497 (20,882,526–24,191,820)	8,324.27 (7,782.26–8,871.02)	0.06 (0.04–0.07)
Southern Sub-Saharan Africa	2,945,199 (2,740,170–3,170,275)	8,946.44 (8,370.42–9,563.71)	5,972,951 (5,547,456–6,415,524)	9,037.91 (8,440.6–9,647.06)	0.03 (0.02–0.04)
North Africa and Middle East	19,739,135 (18,244,354–21,279,746)	9,119.72 (8,490.94–9,769.64)	49,668,756 (45,835,433–53,616,786)	9,180.02 (8,523.53–9,830.12)	0.03 (0.02–0.04)
America					
Andean Latin America	1,465,459 (1,365,798–1,575,973)	5,766.55 (5,377.83–6,181.01)	3,743,589 (3,478,926–4,028,408)	5,946.06 (5,524.81–6,371.54)	0.14 (0.12–0.16)
Caribbean	1,867,236 (1,741,663–2,000,227)	6,480.16 (6,037.62–6,925.81)	3,468,705 (3,235,452–3,720,720)	6,632.98 (6,181.25–7,112.38)	0.09 (0.08–0.1)
Central Latin America	8,628,113 (8,065,183–9,238,847)	8,477.01 (7,931.03–9,062.77)	22,101,801 (20,701,763–23,422,153)	8,642.9 (8,089.33–9,163.75)	0.11 (0.09–0.12)
High-income North America	25,125,238 (23,432,071–26,893,288)	7,401.06 (6,920.42–7,900.28)	42,488,578 (39,404,535–45,165,702)	7,434.68 (6,959.33–7,911.44)	0.17 (0.12–0.22)
Southern Latin America	2,695,843 (2,510,441–2,883,801)	5,784.4 (5,397.31–6,182.95)	4,860,631 (4,530,777–5,214,539)	5,970.43 (5,543.08–6,414.98)	0.16 (0.14–0.18)
Tropical Latin America	8,408,163 (7,820,461–9,015,639)	7,763.01 (7,240.67–8,281.35)	19,295,703 (18,022,781–20,680,042)	7,576.23 (7,080.97–8,107.55)	−0.07 (−0.09 to −0.05)
Asia					
Central Asia	5,534,240 (5,182,702–5,905,060)	10,649.08 (10,005.67–11,309.29)	9,356,434 (8,704,146–9,969,948)	10,698.24 (10,022.94–11,348.1)	0.02 (0.01–0.03)
East Asia	69,797,869 (64,858,255–75,190,145)	7,083.56 (6,610.07–7,593.48)	122,849,232 (113,607,491–132,118,615)	6,258.13 (5,823.13–6,729.82)	−0.19 (−0.32 to −0.05)
High-income Asia Pacific	16,878,162 (15,709,632–18,083,496)	8,566.14 (7,979.52–9,169.53)	29,276,226 (27,336,162–31,020,670)	7,920.71 (7,393.74–8,457.44)	−0.28 (−0.32 to −0.24)
South Asia	72,584,182 (67,087,414–78,502,263)	9,936.56 (9,252.61–10,645.69)	158,803,354 (147,190,732–171,556,959)	9,565.26 (8,903.67–10,265.71)	−0.19 (−0.25 to −0.14)
Southeast Asia	33,768,821 (31,068,003–36,689,892)	10,225.21 (9,474.04–11,022.1)	73,561,437 (67,779,796–79,733,436)	10,474.65 (9,718.92–11,301.72)	0.1 (0.09–0.11)
Europe					
Central Europe	8,932,587 (8,363,724–9,525,233)	6,368.64 (5,978.05–6,771.96)	11,481,266 (10,766,159–12,145,281)	6,205.18 (5,841.05–6,596.24)	−0.16 (−0.18 to −0.14)
Eastern Europe	24,173,788 (22,444,048–26,062,971)	9,327.55 (8,680.03–10,050.13)	27,312,843 (25,403,078–29,335,270)	9,266.31 (8,619.37–9,989.49)	−0.02 (−0.03 to −0.02)
Western Europe	29,093,594 (27,230,515–30,911,868)	5,469.9 (5,132.58–5,799.43)	41,591,466 (39,070,950–43,910,754)	5,226.19 (4,924.43–5,544.2)	−0.16 (−0.2 to −0.13)
Oceania					
Australasia	1,399,739 (1,315,064–1,481,429)	6,084.38 (5,719.4–6,456.26)	2,810,777 (2,637,789–2,995,329)	5,910.04 (5,548.15–6,301.57)	−0.06 (−0.08 to −0.04)
Oceania	306,814 (282,467–332,777)	7,742.43 (7,231.44–8,304.17)	776,661 (715,870–846,878)	7,950.68 (7,408.82–8,567.73)	0.12 (0.1–0.14)
Socio-Demographic index					
High SDI	72,796,005 (68,039,542–77,665,594)	6,883.99 (6,452.03–7,322.72)	120,879,407 (113,333,443–127,967,827)	6,733.55 (6,322.09–7,159.65)	−0.03 (−0.07 to 0.01)
High-middle SDI	78,208,075 (72,803,156–84,086,890)	7,641.21 (7,149.96–8,188.95)	127,111,061 (118,337,580–136,309,239)	7,267.74 (6,782.08–7,816.7)	−0.09 (−0.14 to −0.04)
Middle SDI	105,819,342 (98,201,190–113,755,650)	8,450.59 (7,891.9–9,069.19)	220,823,054 (205,500,732–237,345,634)	8,280.06 (7,728.02–8,885.29)	0.01 (−0.03 to 0.05)
Low-middle SDI	70,588,690 (65,338,440–76,043,937)	9,292.53 (8,660.54–9,952.48)	149,861,034 (139,108,422–161,539,154)	9,171.03 (8,543.99–9,848.15)	−0.07 (−0.1 to −0.05)
Low SDI	23,236,078 (21,506,138–25,034,232)	8,090.37 (7,566.41–8,624.95)	54,528,960 (50,362,040–59,039,888)	7,984.37 (7,449.39–8,549.73)	−0.07 (−0.09 to −0.05)

ASPR, age-standardized prevalence rate; CI, confidence interval; CKD, chronic kidney disease; EAPC, estimated annual percentage change; SDI, socio-demographic index; UI, uncertainty interval.

**Table 2:** Global CKD incidence by SDI and regions in 1990 and 2021, as well as changes in trends from 1990 to 2021.

Location	1990		2021		1990–2021 EAPC, No. (95%CI)
	Incidence cases (95%UI)	ASIR (Per 100,000 population)	Incidence cases (95%UI)	ASIR (Per 100,000 population)	
Sex					
Female	4,405,633 (4,083,071–4,760,087)	201.88 (187.73–218.45)	11,128,691 (10,445,862–11,806,798)	246.03 (231.84–260.67)	0.65 (0.64–0.66)
Male	3,385,072 (3,133,504–3,642,480)	180.89 (167.97–195.41)	8,806,346 (8,248,899–9,359,371)	220.11 (207.07–233.07)	0.64 (0.63–0.66)
Global	7,790,705 (7,226,165–8,402,568)	192.16 (178.69–207.34)	19,935,038 (18,702,793–21,170,794)	233.56 (220.02–247.24)	0.64 (0.63–0.65)
Region					
Africa					
Central Sub-Saharan Africa	28,155 (26,057–30,337)	94.53 (86.79–103.02)	88,402 (82,071–94,885)	130.46 (120.3–142.08)	0.99 (0.84–1.14)
Eastern Sub-Saharan Africa	94,221 (87,688–101,006)	94.88 (87.6–102.96)	244,189 (228,098–260,057)	118.08 (108.97–127.55)	0.6 (0.47–0.73)
Western Sub-Saharan Africa	155,997 (145,849–167,785)	136.02 (125.9–147.1)	455,812 (426,702–486,021)	179.77 (165.43–194.2)	0.89 (0.77–1)
Southern Sub-Saharan Africa	52,502 (48,770–56,528)	169.07 (156.5–183.9)	142,811 (132,638–152,029)	233.6 (216.73–248.74)	0.96 (0.83–1.1)
North Africa and Middle East	482,950 (445,183–524,328)	255.33 (235.33–277.99)	1,988,923 (1,863,403–2,127,840)	411.19 (385.4–438.45)	1.5 (1.43–1.57)
America					
Andean Latin America	36,900 (33,960–40,277)	161.47 (147.69–177.34)	177,934 (165,435–192,798)	298.18 (276.18–322.6)	2.27 (2.17–2.38)
Caribbean	46,852 (43,426–50,526)	168.79 (155.99–183.62)	145,322 (136,425–154,427)	274.22 (257.52–291.86)	1.71 (1.61–1.8)
Central Latin America	266,579 (245,613–291,023)	275.61 (252.21–302.08)	1,056,443 (1,000,773–1,110,306)	411.41 (390.17–431.32)	1.43 (1.36–1.5)
High-income North America	1,050,737 (962,035–1,145,711)	299.22 (274.55–324.91)	2,026,546 (1,868,598–2,175,327)	316.15 (294.31–338.42)	0.1 (0.02–0.17)
Southern Latin America	97,990 (89,314–107,374)	212.82 (194.87–232.31)	244,517 (224,582–263,090)	281.73 (260.45–302.55)	1.08 (0.96–1.19)
Tropical Latin America	194,244 (179,335–210,104)	192.83 (176.83–209.53)	663,468 (620,124–707,786)	259.33 (242.85–275.61)	0.91 (0.86–0.96)
Asia					
Central Asia	64,439 (59,475–69,705)	114.2 (105.1–124.85)	168,442 (154,020–183,300)	187.28 (173.8–201.56)	1.75 (1.61–1.9)
East Asia	1,331,855 (1,207,022–1,453,259)	149.1 (135.79–163.09)	3,505,756 (3,245,783–3,750,967)	166.6 (156.01–176.8)	0.52 (0.46–0.59)
High-income Asia Pacific	538,465 (494,808–584,025)	269.95 (249.25–291.71)	1,249,340 (1,151,298–1,346,531)	274.49 (255.82–293.59)	0.11 (0.07–0.16)
South Asia	1,016,075 (938,359–1,099,033)	146.23 (134.74–158.66)	2,755,183 (2,545,113–2,967,871)	177.65 (164.06–192.03)	0.44 (0.36–0.52)
Southeast Asia	466,174 (432,763–502,980)	156.35 (144.87–169.32)	1,568,475 (1,457,741–1,685,245)	231.28 (215.53–247.34)	1.26 (1.23–1.3)
Europe					
Central Europe	193,169 (174,647–213,352)	132.33 (120.8–145.32)	458,516 (423,618–495,453)	218.39 (203.44–234.06)	1.5 (1.38–1.62)
Eastern Europe	277,461 (253,751–306,742)	108.38 (100.3–117.98)	548,051 (501,351–595,627)	180.1 (167.54–193.25)	1.7 (1.64–1.77)
Western Europe	1,328,467 (1,221,423–1,459,692)	223.93 (207.15–244.09)	2,271,237 (2,122,275–2,425,621)	238.55 (223.94–254.27)	0.25 (0.21–0.3)
Oceania					
Australasia	62,421 (59,023–66,438)	259.18 (245.73–274.3)	160,661 (146,984–172,346)	297.3 (274.48–317.56)	0.45 (0.4–0.51)
Oceania	5,052 (4,665–5,452)	127.57 (117.55–138.5)	15,011 (13,905–16,088)	162.83 (151.06–174.45)	0.75 (0.68–0.81)
Socio-Demographic index					
High SDI	2,789,648 (2,576,788–3,024,433)	252.3 (234.15–272.41)	5,666,653 (5,273,251–6,033,381)	277.75 (260.7–295.01)	0.28 (0.24–0.32)
High-middle SDI	1,574,444 (1,447,925–1,709,190)	160.44 (148.12–174.09)	3,917,149 (3,653,537–4,175,391)	205.9 (194.1–218.61)	0.92 (0.89–0.95)
Middle SDI	1,963,348 (1,808,277–2,127,370)	171.16 (157.68–185.82)	6,251,147 (5,866,964–6,655,539)	232.96 (219.53–246.12)	1.06 (1.04–1.08)
Low-middle SDI	1,107,201 (1,026,640–1,198,009)	153.07 (141.43–166.4)	3,130,960 (2,912,350–3,362,301)	204.97 (189.81–220.29)	0.82 (0.76–0.88)
Low SDI	348,934 (324,143–375,458)	121.74 (112.82–132.3)	950,557 (888,494–1,011,543)	155 (143.4–167.34)	0.7 (0.62–0.78)

ASIR, age-standardized incidence rate; CI, confidence interval; CKD, chronic kidney disease; EAPC, estimated annual percentage change; SDI, socio-demographic index; UI, uncertainty interval.

**Table 3:** Global CKD morality by SDI and regions in 1990 and 2021, as well as changes in trends from 1990 to 2021.

Location	1990		2021		1990–2021 EAPC, No. (95%CI)
	Death cases (95%UI)	ASMR (Per 100,000 population)	Death cases (95%UI)	ASMR (Per 100,000 population)	
Sex					
Female	261,007 (237,127–287,997)	12.64 (11.44–14)	733,120 (654,830–795,633)	16.9 (14.22–17.27)	0.83 (0.75–0.9)
Male	291,666 (259,303–334,444)	18.13 (16.26–21.03)	794,519 (719,354–856,326)	21.91 (19.66–23.60)	0.74 (0.68–0.8)
Global	552,673 (513,463–607,915)	14.85 (13.64–16.38)	1,527,639 (1,389,377–1,638,914)	18.5 (16.72–19.85)	0.82 (0.75–0.89)
Region					
Africa					
Central Sub-Saharan Africa	8,667 (7,200–10,419)	42.68 (35.55–51.04)	21,011 (16,114–27,467)	43.69 (33.26–56.29)	−0.03 (−0.12 to 0.06)
Eastern Sub-Saharan Africa	30,615 (26,919–35,172)	42.4 (37.14–50.19)	60,918 (53,817–69,935)	40.09 (35.57–46.03)	−0.36 (−0.44 to −0.28)
Western Sub-Saharan Africa	28,294 (24,259–32,687)	33.65 (28.97–39.19)	64,796 (53,512–76,124)	36.4 (31.14–42.75)	0.2 (0.16–0.24)
Southern Sub-Saharan Africa	5,376 (4,674–6,619)	20.86 (17.97–26.16)	17,365 (15,612–19,485)	34.43 (31.05–38.29)	1.76 (1.39–2.14)
North Africa and Middle East	45,472 (37,703–66,491)	31.22 (25.48–47.04)	144,687 (126,153–162,699)	37.71 (32.73–42.39)	0.79 (0.64–0.95)
America					
Andean Latin America	5,795 (5,288–6,456)	28.53 (25.87–31.87)	21,619 (17,903–25,800)	37.66 (31.27–44.93)	0.86 (0.54–1.18)
Caribbean	4,754 (4,399–5,437)	18.73 (17.3–21.32)	13,888 (12,086–16,193)	25.78 (22.39–30.07)	1.48 (1.34–1.63)
Central Latin America	22,182 (21,513–22,820)	27.94 (26.82–28.83)	104,444 (94,171–116,461)	42.35 (38.32–47.03)	1.81 (1.34–2.28)
High-income North America	29,949 (27,171–31,433)	8.32 (7.57–8.73)	143,679 (124,827–154,757)	20.55 (18.1–22)	3.4 (3.2–3.6)
Southern Latin America	11,256 (10,698–11,760)	26 (24.55–27.17)	21,583 (19,510–22,986)	23.85 (21.63–25.37)	−0.08 (−0.36 to 0.21)
Tropical Latin America	15,524 (14,832–16,076)	17.94 (16.85–18.75)	46,925 (42,508–49,438)	18.84 (17–19.87)	0.21 (0.06–0.36)
Asia					
Central Asia	2,536 (2,250–2,933)	5.01 (4.38–5.94)	9,397 (8,278–10,515)	12.11 (10.72–13.53)	2.39 (1.91–2.87)
East Asia	107,701 (94,477–126,024)	14.37 (12.68–16.98)	217,342 (178,047–259,057)	11.15 (9.2–13.21)	−0.84 (−0.92 to −0.76)
High-income Asia Pacific	22,233 (20,303–23,333)	12.61 (11.29–13.32)	63,459 (50,432–70,953)	9.74 (8.09–10.68)	−0.92 (−1 to −0.85)
South Asia	79,714 (68,086–89,404)	13.98 (12.14–15.9)	226,043 (192,546–264,155)	16.45 (14.03–19.25)	0.42 (0.3–0.53)
Southeast Asia	57,881 (52,199–66,490)	22.86 (20.59–26.7)	170,033 (148,864–190,537)	28.47 (24.79–31.87)	0.71 (0.66–0.76)
Europe					
Central Europe	14,335 (13,732–14,892)	10.47 (10–10.92)	21,635 (19,445–23,977)	9.39 (8.41–10.47)	−0.29 (−0.45 to −0.14)
Eastern Europe	9,389 (9,183–9,574)	3.58 (3.5–3.65)	17,821 (16,126–19,853)	5.22 (4.73–5.82)	0.67 (0.32–1.02)
Western Europe	48,662 (44,430–50,877)	8.26 (7.52–8.66)	133,481 (109,637–148,166)	10.66 (8.9–11.81)	1.35 (1.19–1.51)
Oceania					
Australasia	1,851 (1,687–1,959)	8.47 (7.66–8.99)	5,968 (5,025–6,525)	9.63 (8.23–10.47)	1.06 (0.82–1.3)
Oceania	489 (342–661)	17.22 (12.83–23.08)	1,546 (1,265–1,892)	21.56 (17.98–26.37)	0.64 (0.53–0.75)
Socio-Demographic index					
High SDI	100,025 (92,113–104,189)	9.22 (8.45–9.62)	340,083 (289,016–369,665)	14.11 (12.3–15.21)	1.73 (1.61–1.86)
High-middle SDI	99,196 (91,758–110,668)	11.36 (10.43–12.65)	226,797 (201,672–252,703)	12.02 (10.68–13.38)	0.25 (0.16–0.34)
Middle SDI	177,302 (162,783–198,065)	19.07 (17.39–21.35)	513,051 (458,865–556,752)	20.89 (18.45–22.67)	0.38 (0.29–0.48)
Low-middle SDI	110,899 (98,721–127,673)	18.59 (16.43–22.23)	309,509 (280,315–349,455)	23.08 (20.97–26.31)	0.71 (0.66–0.75)
Low SDI	64,656 (57,360–73,648)	29.72 (26.31–34.62)	136,797 (118,867–157,579)	29.43 (26.13–33.79)	−0.08 (−0.2 to 0.03)

ASMR, age-standard morality rate; CI, confidence interval; CKD, chronic kidney disease; EAPC, estimated annual percentage change; SDI, socio-demographic index; UI, uncertainty interval.

**Table 4:** Global CKD DALYs by SDI and regions in 1990 and 2021, as well as changes in trends from 1990 to 2021.

Location	1990		2021		1990–2021 EAPC, No. (95%CI)
	DALY cases (95%UI)	ASDR (Per 100,000 population)	DALY cases (95%UI)	ASDR (Per 100,000 population)	
Sex					
Female	9,670,407 (8,788,280–10,592,764)	426.9 (389.12–467.43)	20,693,932 (18,836,614–22,728,832)	465.69 (424.02–511.12)	0.3 (0.25–0.35)
Male	11,069,488 (9,601,627–12,296,840)	546.41 (476.93–612.62)	23,759,752 (21,473,235–26,215,374)	603.4 (546.08–663.35)	0.39 (0.36–0.43)
Global	20,739,895 (18,843,684–22,588,533)	479.85 (439.18–523.79)	44,453,684 (40,840,762–48,508,462)	529.62 (486.25–577.42)	0.37 (0.33–0.41)
Region					
Africa					
Central Sub-Saharan Africa	349,929 (293,393–410,516)	1,158.69 (983.37–1,367.04)	783,700 (622,595–996,532)	1,124.7 (899.03–1,436.09)	−0.19 (−0.26 to −0.12)
Eastern Sub-Saharan Africa	1,117,669 (975,468–1,248,904)	1,095.23 (965.94–1,254.96)	2,047,629 (1,792,019–2,374,164)	948.36 (838.71–1,090.36)	−0.66 (−0.73 to −0.58)
Western Sub-Saharan Africa	1,112,629 (952,298–1,275,995)	928.71 (800.33–1,067.12)	2,437,269 (1,992,652–2,893,649)	930.73 (788.43–1,081.08)	−0.04 (−0.08 to 0)
Southern Sub-Saharan Africa	202,978 (179,955–234,849)	623.85 (548.64–742.1)	551,466 (494,226–622,064)	895.96 (807.34–996.98)	1.32 (0.99–1.65)
North Africa and Middle East	1,493,457 (1,272,351–1,963,220)	759.9 (642.73–1,059.21)	3,925,988 (3,427,227–4,413,622)	846.64 (747.17–948.02)	0.51 (0.41–0.61)
America					
Andean Latin America	190,986 (174,177–211,377)	753.97 (688.02–838.66)	524,246 (434,446–625,013)	872.44 (723.54–1,037.91)	0.39 (0.12–0.67)
Caribbean	161,590 (149,056–180,712)	565.09 (523.55–636.63)	385,286 (331,558–454,109)	735.85 (631.01–867.89)	1.25 (1.13–1.37)
Central Latin America	779,859 (736,752–822,191)	767.86 (724.18–811.62)	2,993,750 (2,691,663–3,371,708)	1,171.14 (1,054.82–1,316.26)	1.75 (1.32–2.18)
High-income North America	895,097 (798,640–986,360)	266.63 (238.28–293.17)	3,120,983 (2,864,207–3,351,576)	508.81 (467.04–545.39)	2.45 (2.3–2.6)
Southern Latin America	281,092 (266,405–294,525)	612.71 (580.9–641.72)	441,138 (411,345–467,913)	515.22 (481.75–546.8)	−0.37 (−0.59 to −0.15)
Tropical Latin America	594,892 (560,161–631,009)	562.72 (527.93–598.67)	1,311,915 (1,220,675–1,401,164)	516.97 (480.55–552.11)	−0.31 (−0.43 to −0.18)
Asia					
Central Asia	176,292 (152,244–202,201)	326.08 (278.46–378.79)	427,633 (377,524–490,696)	493.2 (435.99–565.47)	1.02 (0.68–1.36)
East Asia	4,372,608 (3,843,678–4,996,867)	461.27 (406.84–529.07)	6,486,167 (5,538,060–7,597,380)	322.36 (275.35–377.34)	−1.18 (−1.27 to −1.1)
High-income Asia Pacific	599,581 (550,356–643,830)	315.66 (289.04–339.18)	1,146,613 (990,343–1,268,312)	235.57 (206.04–259.61)	−0.92 (−0.98 to −0.87)
South Asia	3,674,401 (3,157,460–4,115,106)	509.78 (449.63–573.28)	8,443,339 (7,372,315–9,681,828)	540.57 (473.83–620.42)	0.13 (0.08–0.17)
Southeast Asia	2,408,834 (2,133,712–2,690,991)	751.94 (672.31–850.85)	5,703,263 (5,028,657–6,329,025)	846.26 (753.05–940.53)	0.38 (0.35–0.41)
Europe					
Central Europe	469,644 (436,516–504,841)	342.78 (318.66–368.53)	539,982 (472,193–612,672)	266.92 (233.43–306.23)	−0.74 (−0.84 to −0.64)
Eastern Europe	521,705 (463,250–581,791)	207.42 (184.74–230.89)	642,467 (562,943–726,952)	204.68 (180.4–232.13)	−0.58 (−0.77 to −0.38)
Western Europe	1,267,566 (1,125,408–1,409,086)	234.12 (206.76–260.11)	2,361,342 (2,067,098–2,638,163)	241.72 (209.82–271.06)	0.38 (0.29–0.47)
Oceania					
Australasia	46,405 (42,217–50,551)	211.12 (192.43–229.58)	113,693 (101,514–125,301)	216.61 (193.23–239.24)	0.53 (0.36–0.7)
Oceania	22,680 (16,686–29,125)	583.66 (445.79–741.99)	65,815 (55,558–77,796)	699.04 (597.93–821.29)	0.52 (0.42–0.62)
Socio-Demographic index					
High SDI	2,898,450 (2,605,440–3,169,018)	277.64 (250.03–303.24)	7,115,740 (6,464,112–7,759,250)	358.51 (324.74–390.2)	1.07 (0.99–1.16)
High-middle SDI	3,534,406 (3,184,087–3,954,975)	360.84 (325.48–401.53)	5,944,277 (5,372,850–6,623,407)	324.64 (293.58–360.92)	−0.35 (−0.42 to −0.28)
Middle SDI	7,031,758 (6,325,501–7,794,727)	585.56 (531.8–652.64)	15,700,568 (14,206,921–17,147,169)	596.45 (540.33–650.48)	0.12 (0.04–0.2)
Low-middle SDI	4,690,095 (4,056,126–5,163,160)	609.44 (545.07–686.29)	10,611,260 (9,599,821–11,771,755)	686.98 (622.5–765.2)	0.4 (0.38–0.42)
Low SDI	2,564,545 (2,265,146–2,870,454)	853.94 (760.14–971.76)	5,042,405 (4,408,653–5,841,516)	791.8 (704.14–909.1)	−0.34 (−0.42 to −0.25)

ASDR, age-standard DALYs rate; CI, confidence interval; CKD, chronic kidney disease; DALYs, disability-adjusted life years; EAPC, estimated annual percentage change; SDI, socio-demographic index; UI, uncertainty interval.

A non-significant increasing trend was observed in global ASPR data for CKD from 1990 to 2021, with an estimated annual percentage change (EAPC) of 0.01 per 100,000 population annually (95% CI: −0.02 to 0.04). Nevertheless, over the 30-year period, there was a general decline in prevalence (AAPC = −2.1, 95% CI: −3.9% to −0.2%, *p* = 0.030) (Tables 5, 6). Conversely, incidence rates, mortality rates, and DALYs rates have increased significantly over the 30 years. The EAPCs for incidence, mortality, and DALYs rates are 0.64 per 100,000 (95% CI: 0.63–0.65), 0.82 per 100,000 (95% CI: 0.75–0.89), and 0.37 per 100,000 (95% CI: 0.33–0.41). The results indicated a positive trend in the AAPCs for all three indices, with the ASIR (AAPC = 63.4, 95% CI: 62.1–64.6%, *p* < 0.001), ASMR (AAPC = 74.5, 95% CI: 60.3–88.6%, *p* < 0.001), and ASDR (AAPC = 32.2, 95% CI: 26.2–38.3%, *p* < 0.001) indices demonstrating increasing trends (Tables 5, 6).

**Table 5:** Disease burden of CKD and its subtypes by gender, and comparative analysis between 1990 and 2021.

	1990						2021						1990–2021 EAPC, No. (95%CI)		
	Both sexes (95%UI)		Female (95%UI)		Male (95%UI)		Both sexes (95%UI)		Female (95%UI)		Male (95%UI)		Both sexes (95%UI)	Female (95%UI)	Male (95%UI)
	Cases	Rate	Cases	Rate	Cases	Rate	Cases	Rate	Cases	Rate	Cases	Rate			
CKD															
Prevalence	350,962,674 (326,973,785–376,155,723)	8,072.75 (7,560.37–8,634.07)	188,185,026 (175,770,189–201,580,675)	8,293.08 (7,778.75–8,870.27)	162,777,648 (151,189,910–174,916,268)	7,812.8 (7,301.88–8,356.28)	673,722,703 (629,095,119–722,364,096)	8,006 (7,482.12–8,575.62)	358,777,424 (335,684,717–383,643,812)	8,182.65 (7,653.14–8,764.76)	314,945,280 (293,385,891–338,118,606)	7,808.96 (7,288.71–8,366.6)	0.01 (−0.02 to 0.04)	0 (−0.04 to 0.03)	0.03 (0–0.05)
Incidence	7,790,705 (7,226,165–8,402,568)	192.16 (178.69–207.34)	4,405,633 (4,083,071–4,760,087)	201.88 (187.73–218.45)	3,385,072 (3,133,504–3,642,480)	180.89 (167.97–195.41)	19,935,038 (18,702,793–21,170,794)	233.56 (220.02–247.24)	11,128,691 (10,445,862–11,806,798)	246.03 (231.84–260.67)	8,806,346 (8,248,899–9,359,371)	220.11 (207.07–233.07)	0.64 (0.63–0.65)	0.65 (0.64–0.66)	0.64 (0.63–0.66)
Deaths	552,673 (513,463–607,915)	14.85 (13.64–16.38)	261,007 (237,127–287,997)	12.64 (11.44–13.99)	291,666 (259,303–334,444)	18.13 (16.26–21.03)	1,527,639 (1,389,377–1,638,914)	18.5 (16.72–19.85)	733,120 (654,830–795,633)	15.9 (14.22–17.27)	794,519 (719,354–856,326)	21.91 (19.66–23.6)	0.82 (0.75–0.89)	0.83 (0.75–0.9)	0.74 (0.68–0.8)
DALYs	20,739,895 (18,843,684–22,588,533)	479.85 (439.18–523.79)	9,670,407 (8,788,280–10,592,764)	426.9 (389.12–467.43)	11,069,488 (9,601,627–12,296,840)	546.41 (476.93–612.62)	44,453,684 (40,840,762–48,508,462)	529.62 (486.25–577.42)	20,693,932 (18,836,614–22,728,832)	465.69 (424.02–511.12)	23,759,752 (21,473,235–26,215,374)	603.4 (546.08–663.35)	0.37 (0.33–0.41)	0.3 (0.25–0.35)	0.39 (0.36–0.43)
T1DM															
Prevalence	49,300 (39,088–61,208)	1.08 (0.84–1.35)	22,377 (17,833–28,820)	0.97 (0.77–1.26)	26,924 (20,680–34,089)	1.19 (0.9–1.53)	94,020 (71,457–119,984)	1.08 (0.83–1.38)	39,416 (30,272–51,111)	0.89 (0.69–1.15)	54,604 (40,656–69,965)	1.28 (0.96–1.63)	−0.07 (−0.13 to 0)	−0.42 (−0.52 to −0.33)	0.2 (0.16–0.25)
Incidence	2,967,857 (2,607,069–3,328,285)	57.54 (50.87–64.11)	1,728,367 (1,482,021–1,974,137)	66.85 (57.8–75.9)	1,239,490 (1,113,261–1,373,486)	48.37 (43.74–53.53)	6,295,711 (5,459,693–7,114,345)	77.31 (66.91–87.58)	3,668,687 (3,109,394–4,232,810)	90.71 (76.97–104.6)	2,627,023 (2,318,287–2,923,705)	64.19 (56.78–71.74)	1.35 (1.25–1.46)	1.37 (1.27–1.47)	1.33 (1.22–1.45)
Deaths	63,601 (52,476–76,375)	1.1 (0.92–1.31)	26,773 (21,783–33,119)	0.94 (0.77–1.15)	36,829 (30,520–43,841)	1.25 (1.06–1.48)	95,140 (82,237–111,471)	1.31 (1.12–1.55)	38,228 (32,027–45,888)	1.08 (0.89–1.33)	56,911 (49,528–65,500)	1.53 (1.32–1.78)	0.69 (0.63–0.74)	0.55 (0.51–0.58)	0.79 (0.73–0.86)
DALYs	2,227,518 (1,835,373–2,679,208)	47.05 (38.4–57.32)	1,010,830 (828,455–1,248,632)	42.69 (34.68–53.34)	1,216,689 (978,326–1,486,172)	51.44 (40.65–63.6)	3,875,628 (3,062,396–4,845,503)	45.2 (36.01–56.35)	1,609,247 (1,278,069–2,036,183)	37.27 (29.68–47.09)	2,266,380 (1,739,578–2,862,625)	53.21 (40.96–67.05)	−0.21 (−0.28 to −0.14)	−0.59 (−0.69 to −0.49)	0.08 (0.03–0.13)
T2DM															
Prevalence	58,105,268 (53,056,992–63,286,818)	1,327.22 (1,223.26–1,439.42)	28,360,560 (25,996,835–30,841,479)	1,250.08 (1,152.38–1,357.8)	29,744,708 (27,057,208–32,317,657)	1,414.09 (1,298.44–1,528.78)	107,559,955 (99,170,797–115,994,732)	1,259.63 (1,161.99–1,359.92)	52,837,532 (48,769,234–56,931,920)	1,191.96 (1,100.99–1,286.63)	54,722,423 (50,395,175–59,068,229)	1,333.1 (1,229.35–1,437.39)	−0.17 (−0.2 to −0.13)	−0.14 (−0.19 to −0.09)	−0.21 (−0.23 to −0.18)
Incidence	753,106 (680,930–826,928)	19.07 (17.28–20.83)	376,968 (341,204–413,084)	17.62 (15.96–19.26)	376,138 (339,359–412,959)	20.88 (18.91–22.8)	2,012,025 (1,857,800–2,154,288)	23.07 (21.4–24.72)	990,074 (915,628–1,063,729)	21.42 (19.84–22.97)	1,021,951 (943,498–1,098,453)	25.01 (23.14–26.82)	0.61 (0.6–0.62)	0.63 (0.62–0.65)	0.57 (0.56–0.58)
Deaths	147,970 (124,179–176,413)	4.15 (3.5–4.94)	72,522 (60,489–86,024)	3.6 (3–4.29)	75,448 (61,702–93,510)	4.98 (4.09–6.13)	477,273 (401,541–565,951)	5.72 (4.83–6.79)	230,668 (193,896–274,409)	4.93 (4.15–5.86)	246,605 (204,152–294,508)	6.77 (5.57–8.14)	1.16 (1.09–1.24)	1.1 (1.02–1.17)	1.16 (1.09–1.23)
DALYs	4,122,919 (3,498,980–4,818,958)	105.71 (90.68–122.67)	1,975,646 (1,674,230–2,307,158)	93.54 (79.27–108.89)	2,147,273 (1,750,418–2,594,132)	121.65 (100.4–145.91)	11,278,935 (9,682,785–13,103,871)	131.08 (112.75–152.49)	5,262,931 (4,502,103–6,118,770)	113.67 (97.44–132.05)	6,016,004 (5,067,209–7,070,255)	151.81 (129.54–177.25)	0.8 (0.74–0.87)	0.69 (0.63–0.76)	0.86 (0.8–0.92)
Glomerulonephritis															
Prevalence	244,229 (218,811–271,857)	4.3 (3.88–4.75)	87,235 (77,100–99,306)	3.1 (2.75–3.5)	156,994 (141,222–173,481)	5.48 (4.98–6.01)	357,288 (329,226–388,483)	4.84 (4.42–5.29)	120,654 (109,596–133,046)	3.38 (3.04–3.77)	236,634 (218,787–254,836)	6.28 (5.77–6.78)	0.39 (0.36–0.43)	0.28 (0.24–0.32)	0.45 (0.41–0.49)
Incidence	6,370,882 (5,926,723–6,848,154)	128.55 (119.33–137.58)	2,644,789 (2,434,847–2,883,624)	106.92 (98.49–116.09)	3,726,093 (3,484,081–3,986,630)	150.9 (141.25–160.98)	10,735,809 (9,925,500–11,520,171)	129.94 (120.25–139.51)	4,531,261 (4,169,272–4,956,553)	108.5 (99.78–118.56)	6,204,548 (5,774,782–6,630,226)	151.85 (141.58–162.39)	0.06 (0.04–0.08)	0.09 (0.06–0.11)	0.04 (0.02–0.06)
Deaths	83,170 (69,625–97,738)	2.02 (1.68–2.38)	37,141 (31,114–43,462)	1.67 (1.38–1.96)	46,028 (37,748–54,972)	2.5 (2.05–3.01)	193,997 (162,332–226,569)	2.34 (1.96–2.74)	89,022 (73,524–105,883)	1.99 (1.66–2.36)	104,975 (87,280–123,438)	2.78 (2.3–3.26)	0.54 (0.5–0.59)	0.6 (0.54–0.66)	0.43 (0.39–0.47)
DALYs	3,751,088 (3,252,292–4,269,460)	77.78 (67.62–88.41)	1,655,577 (1,429,752–1,885,529)	67.19 (57.78–76.73)	2,095,512 (1,783,982–2,409,288)	90.23 (77.22–104.71)	6,959,758 (6,018,414–7,961,673)	84.47 (73.2–96.13)	3,068,936 (2,640,502–3,521,006)	72.47 (62.8–82.98)	3,890,822 (3,322,946–4,499,291)	97.57 (83.48–112.8)	0.28 (0.25–0.31)	0.23 (0.19–0.28)	0.29 (0.27–0.31)
Hypertension															
Prevalence	11,712,345 (10,891,658–12,623,876)	310.68 (289.07–333.84)	5,934,028 (5,515,951–6,381,488)	285.32 (265.67–306.5)	5,778,317 (5,369,612–6,220,947)	344.62 (320.69–370.53)	24,467,653 (22,861,634–26,230,869)	291.19 (272.49–311.88)	11,975,406 (11,204,542–12,802,395)	261.49 (244.79–279.02)	12,492,247 (11,676,417–13,414,331)	327.71 (306.18–351.39)	−0.16 (−0.18 to −0.13)	−0.22 (−0.25 to −0.19)	−0.12 (−0.14 to −0.1)
Incidence	463,924 (426,189–505,831)	12.24 (11.31–13.33)	235,211 (216,325–257,276)	11.23 (10.36–12.21)	228,713 (209,258–248,913)	13.59 (12.56–14.86)	1,282,205 (1,195,230–1,366,296)	14.97 (14.02–15.93)	633,339 (591,756–672,358)	13.71 (12.82–14.56)	648,865 (605,404–692,260)	16.54 (15.47–17.64)	0.66 (0.65–0.67)	0.66 (0.65–0.66)	0.64 (0.64–0.65)
Deaths	148,983 (123,167–176,985)	4.29 (3.55–5.11)	68,301 (56,331–80,968)	3.47 (2.85–4.11)	80,682 (65,428–100,070)	5.53 (4.55–6.86)	454,359 (381,291–524,688)	5.54 (4.68–6.41)	214,533 (175,442–249,402)	4.6 (3.77–5.33)	239,825 (200,045–280,262)	6.8 (5.66–7.85)	0.96 (0.9–1.02)	1.03 (0.96–1.11)	0.82 (0.76–0.87)
DALYs	4,344,896 (3,676,494–5,110,004)	107.77 (91.26–126.92)	1,915,563 (1,616,163–2,234,955)	88.81 (74.66–103.46)	2,429,333 (2,032,444–2,969,455)	132.24 (111.27–160.76)	10,850,728 (9,207,080–12,320,650)	128.41 (109.14–145.64)	4,814,732 (4,100,512–5,513,964)	106.04 (90.33–121.44)	6,035,995 (5,059,223–7,010,183)	154.81 (130.79–178.18)	0.62 (0.58–0.67)	0.6 (0.54–0.66)	0.59 (0.55–0.63)
Other															
Prevalence	271,806,360 (253,122,975–291,694,859)	6,248.77 (5,852.52–6,683.27)	149,517,320 (139,624,963–160,490,353)	6,583.91 (6,168.11–7,039.66)	122,289,039 (113,501,069–131,644,298)	5,854.82 (5,470.99–6,265.45)	524,663,972 (488,899,870–562,780,408)	6,247.94 (5,823.88–6,691.38)	285,764,930 (267,230,946–305,356,489)	6,530.01 (6,090.76–6,986.52)	238,899,041 (221,964,877–257,251,009)	5,932.11 (5,522.85–6,362.71)	0.04 (0.01–0.07)	0.01 (−0.02 to 0.05)	0.08 (0.05–0.1)
Incidence	6,265,844 (5,808,707–6,762,739)	155.45 (144.13–167.87)	3,679,445 (3,411,383–3,983,479)	169 (156.95–182.99)	2,586,399 (2,387,039–2,788,824)	139.68 (129.2–150.66)	16,188,381 (15,192,359–17,180,106)	189.36 (178.21–200.61)	9,346,396 (8,784,299–9,915,713)	206.44 (194.9–218.84)	6,841,985 (6,404,404–7,271,000)	170.74 (160.45–180.96)	0.65 (0.64–0.66)	0.66 (0.65–0.67)	0.66 (0.65–0.67)
Deaths	123,249 (104,874–140,205)	3.32 (2.78–3.82)	60,666 (51,157–68,834)	2.93 (2.47–3.34)	62,584 (52,112–73,492)	3.93 (3.17–4.74)	307,990 (259,417–357,326)	3.81 (3.2–4.41)	159,480 (131,659–185,890)	3.48 (2.91–4.04)	148,510 (124,115–174,016)	4.29 (3.6–5)	0.59 (0.5–0.69)	0.7 (0.6–0.81)	0.41 (0.33–0.48)
DALYs	6,293,473 (5,433,783–7,066,172)	141.53 (122.79–160.33)	3,112,792 (2,699,263–3,559,928)	134.66 (116.65–153.58)	3,180,681 (2,619,213–3,612,428)	150.85 (126.07–171.29)	11,488,636 (10,007,838–13,046,210)	140.45 (122.56–159.28)	5,938,086 (5,122,641–6,815,624)	136.24 (117.73–155.85)	5,550,550 (4,837,739–6,316,302)	146 (127.54–166.15)	0.03 (−0.01 to 0.07)	0.09 (0.05–0.13)	−0.05 (−0.09 to −0.01)

CI, confidence interval; CKD, chronic kidney disease; DALYs, disability-adjusted life years; EAPC, estimated annual percentage change; SDI, socio-demographic index; T1DM, type 1 diabetes mellitus; T2DM, type 2 diabetes mellitus; UI, uncertainty interval.

**Table 6:** Global average annual percentage changes in the burden of CKD and its subtypes.

Location measure	CKD		T1DM		T2DM		Glomerulonephritis		Hypertension		Other	
	AAPC (95%CI)	*p*-value	AAPC (95%CI)	*p*-value	AAPC (95%CI)	*p*-value	AAPC (95%CI)	*p*-value	AAPC (95%CI)	*p*-value	AAPC (95%CI)	*p*-value
Prevalence												
Global	−0.021 (−0.039 to −0.002)	0.030	0.956 (0.847–1.065)	<0.001	−0.169 (−0.197 to −0.14)	<0.001	0.041 (0.027–0.054)	<0.001	−0.213 (−0.244 to −0.181)	<0.001	0.007 (−0.016 to 0.031)	0.538
Africa	0.032 (0.027–0.037)	<0.001	0.47 (0.367–0.574)	<0.001	−0.101 (−0.112 to −0.089)	<0.001	−0.004 (−0.019 to 0.01)	0.566	−0.102 (−0.107 to −0.097)	<0.001	0.062 (0.05–0.073)	<0.001
America	0.045 (0.009–0.081)	0.015	0.432 (0.378–0.487)	<0.001	0.005 (−0.047 to 0.058)	0.844	0.17 (0.126–0.214)	<0.001	0.109 (0.076–0.142)	<0.001	0.037 (0.008–0.066)	0.012
Asia	−0.105 (−0.125 to −0.085)	<0.001	1.129 (1.032–1.226)	<0.001	−0.273 (−0.33 to −0.216)	<0.001	0.023 (0.006–0.04)	0.009	−0.4 (−0.487 to −0.314)	<0.001	−0.068 (−0.088 to −0.047)	<0.001
Europe	−0.073 (−0.08 to −0.066)	<0.001	1.833 (1.779–1.888)	<0.001	−0.303 (−0.32 to −0.286)	<0.001	−0.163 (−0.18 to −0.146)	<0.001	−0.165 (−0.181 to −0.149)	<0.001	−0.056 (−0.062 to −0.05)	<0.001
Oceania	0.087 (0.077–0.096)	<0.001	0.137 (0.073–0.202)	<0.001	−0.143 (−0.158 to −0.127)	<0.001	0.161 (0.151–0.171)	<0.001	0.012 (−0.006 to 0.03)	0.200	0.141 (0.129–0.153)	<0.001
Incidence												
Global	0.634 (0.621–0.646)	<0.001	0.59 (0.537–0.643)	<0.001	0.617 (0.598–0.637)	<0.001	0.394 (0.361–0.427)	<0.001	0.654 (0.638–0.669)	<0.001	0.641 (0.628–0.654)	<0.001
Africa	1.262 (1.2–1.324)	<0.001	0.532 (0.495–0.569)	<0.001	1.331 (1.284–1.377)	<0.001	0.625 (0.583–0.667)	<0.001	1.325 (1.262–1.388)	<0.001	1.285 (1.185–1.385)	<0.001
America	0.619 (0.604–0.633)	<0.001	0.625 (0.566–0.683)	<0.001	0.619 (0.607–0.631)	<0.001	0.665 (0.653–0.678)	<0.001	0.588 (0.578–0.598)	<0.001	0.62 (0.607–0.634)	<0.001
Asia	0.607 (0.59–0.625)	<0.001	0.453 (0.37–0.536)	<0.001	0.517 (0.495–0.539)	<0.001	0.25 (0.178–0.322)	<0.001	0.683 (0.663–0.702)	<0.001	0.625 (0.602–0.647)	<0.001
Europe	0.847 (0.832–0.862)	<0.001	1.052 (0.951–1.154)	<0.001	0.885 (0.866–0.905)	<0.001	0.219 (0.197–0.241)	<0.001	0.882 (0.846–0.918)	<0.001	0.852 (0.839–0.866)	<0.001
Oceania	0.788 (0.757–0.819)	<0.001	0.3 (0.263–0.338)	<0.001	0.8 (0.743–0.857)	<0.001	0.427 (0.377–0.477)	<0.001	0.889 (0.862–0.916)	<0.001	0.8 (0.771–0.829)	<0.001
Death												
Global	0.745 (0.603–0.886)	<0.001	0.026 (−0.087 to 0.138)	0.655	1.069 (0.928–1.209)	<0.001	0.499 (0.412–0.587)	<0.001	0.868 (0.779–0.957)	<0.001	0.468 (0.344–0.593)	<0.001
Africa	0.513 (0.46–0.566)	<0.001	−0.317 (−0.378 to −0.256)	<0.001	0.538 (0.497–0.579)	<0.001	0.145 (0.071–0.219)	<0.001	0.623 (0.567–0.678)	<0.001	0.777 (0.726–0.829)	<0.001
America	1.835 (1.663–2.008)	<0.001	1.713 (1.478–1.948)	<0.001	2.583 (2.441–2.725)	<0.001	1.534 (1.223–1.847)	<0.001	1.844 (1.677–2.011)	<0.001	0.849 (0.537–1.162)	<0.001
Asia	0.07 (−0.051–0.191)	0.259	−0.495 (−0.567 to −0.423)	<0.001	0.172 (0.009–0.335)	0.039	−0.009 (−0.155 to 0.138)	0.908	0.108 (−0.034 to 0.251)	0.137	0.048 (−0.078 to 0.175)	0.456
Europe	0.87 (0.54–1.201)	<0.001	−0.178 (−0.727 to 0.374)	0.526	1.238 (0.968–1.509)	<0.001	0.344 (−0.023 to 0.714)	0.067	1.217 (0.786–1.651)	<0.001	0.858 (0.604–1.114)	<0.001
Oceania	0.715 (0.57–0.86)	<0.001	0.664 (0.587–0.742)	<0.001	0.77 (0.64–0.9)	<0.001	0.717 (0.628–0.806)	<0.001	0.585 (0.447–0.723)	<0.001	0.724 (0.588–0.86)	<0.001
DALYs												
Global	0.322 (0.262–0.383)	<0.001	−0.119 (−0.209 to −0.03)	0.009	0.696 (0.595–0.797)	<0.001	0.271 (0.171–0.37)	<0.001	0.583 (0.481–0.684)	<0.001	−0.016 (−0.088 to 0.056)	0.655
Africa	0.198 (0.152–0.244)	<0.001	−0.345 (−0.383 to −0.308)	<0.001	0.282 (0.247–0.317)	<0.001	−0.064 (−0.112 to −0.016)	0.009	0.434 (0.327–0.542)	<0.001	0.246 (0.172–0.319)	<0.001
America	1.333 (1.089–1.577)	<0.001	1.421 (1.209–1.633)	<0.001	2.053 (1.847–2.259)	<0.001	1.091 (0.815–1.367)	<0.001	1.572 (1.394–1.75)	<0.001	0.431 (0.233–0.63)	<0.001
Asia	−0.19 (−0.272 to −0.108)	<0.001	−0.573 (−0.646 to −0.5)	<0.001	0.006 (−0.091 to 0.103)	0.899	−0.231 (−0.308 to −0.155)	<0.001	0.008 (−0.063 to 0.079)	0.833	−0.372 (−0.462 to −0.283)	<0.001
Europe	0.025 (−0.193 to 0.244)	0.820	−0.1 (−0.528 to 0.33)	0.648	0.502 (0.309–0.696)	<0.001	−0.466 (−0.756 to −0.175)	0.002	0.504 (0.262–0.747)	<0.001	−0.091 (−0.213 to 0.031)	0.145
Oceania	0.623 (0.541–0.706)	<0.001	0.612 (0.523–0.701)	<0.001	0.695 (0.562–0.828)	<0.001	0.562 (0.489–0.636)	<0.001	0.458 (0.355–0.562)	<0.001	0.385 (0.242–0.528)	<0.001

AAPC, average annual percentage change; CI, confidence interval; CKD, chronic kidney disease; DALYs, disability-adjusted life years; T1DM, type 1 diabetes mellitus; T2DM, type 2 diabetes mellitus; UI, uncertainty interval.

### Regional level

**Figure 2:**
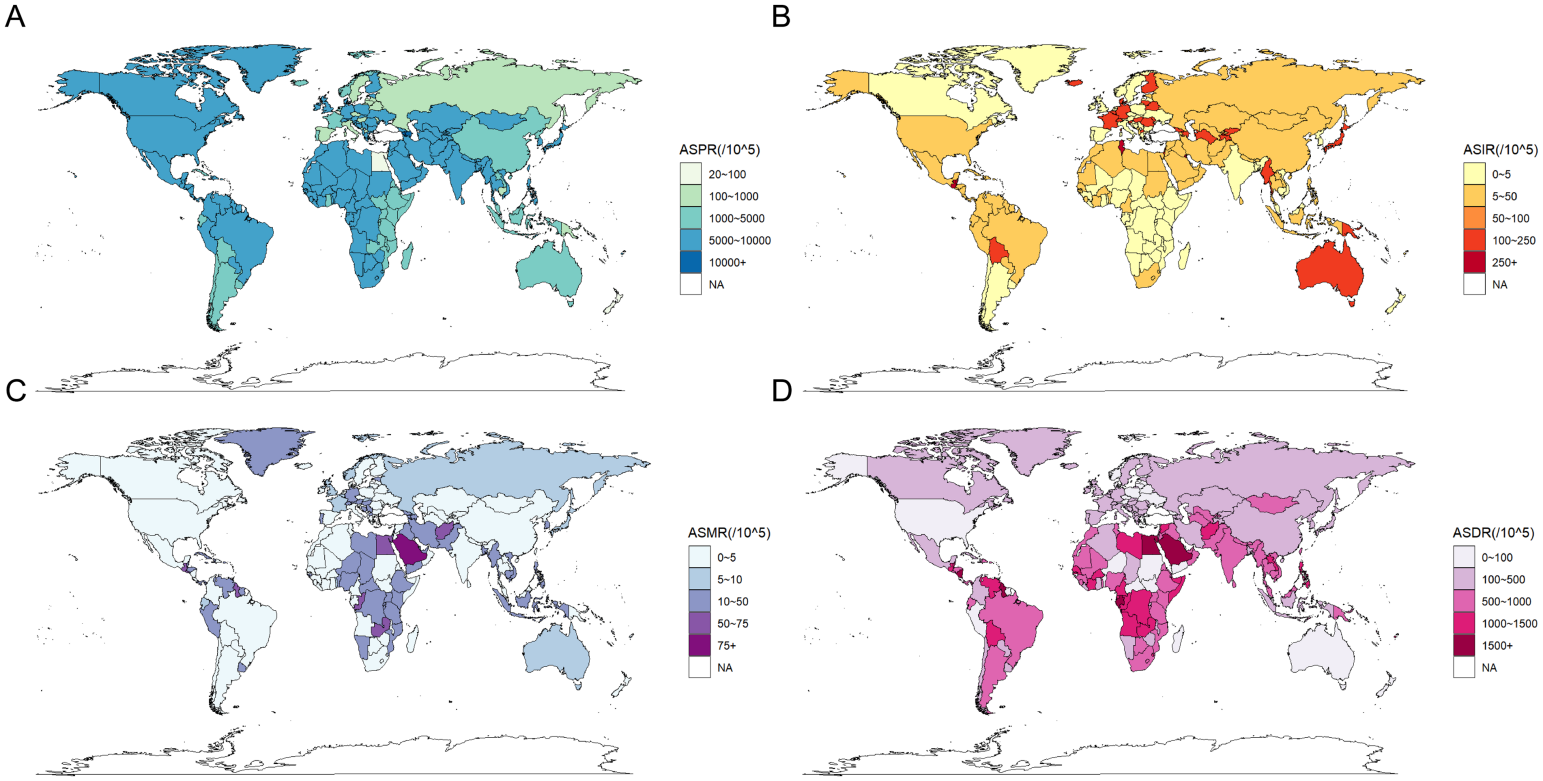
Global distribution of the burden of chronic kidney disease in 2021. **(A)** Age standardized prevalence rate (per 100,000 population), both sexes; **(B)** Age standardized incidence rate (per 100,000 population), both sexes; **(C)** Age standardized mortality rate (per 100,000 population), both sexes; **(D)** Age standardized disability-adjusted life rate (per 100,000 population), both sexes.

In 2021, the ASPR of CKD was highest in Central Asia at 10,698.24 per 100,000 population (95% UI: 10,022.94–11,348.1), followed by Southeast Asia at 10,474.65 per 100,000 population (95% UI: 9,718.92–11,301.72). Concurrently, the highest ASPR on a global SDI level was found in low-middle SDI regions at 9,171.03 per 100,000 population (95% UI: 8,543.99–9,848.15). In contrast, regions with a high SDI exhibited the lowest ASPR at 6,733.55 per 100,000 population (95% UI: 6,322.09–7,159.65), with Western Europe at 5,226.19 per 100,000 population (95% UI: 4,924.43–5,544.2) and Australasia at 5,910.04 per 100,000 population (95% UI: 5,548.15–6,301.57) (Table 1; Figure 2A).

A comparison of the ASPR variations across different global regions over the period from 1990 to 2021 revealed distinct trends over the 30-year period. Geographically, the highest income North America exhibited the most significant increase, with an EAPC of 0.17 per 100,000 population (95% CI: 0.12–0.22). This trend was followed by Southern Latin America, which had an EAPC of 0.16 per 100,000 population (95% CI: 0.14–0.18), and Andean Latin America, with an EAPC of 0.14 per 100,000 population (95% CI: 0.12–0.16). In contrast, the most significant decrease was observed in high-income Asia Pacific, with an EAPC of −0.28 per 100,000 population (95% CI: −0.32 to −0.24). This was followed by East Asia, with an EAPC of −0.19 per 100,000 population (95% CI: −0.32 to −0.05), and South Asia, with an EAPC of −0.19 per 100,000 population (95% UI: −0.25 to −0.14). On a global SDI level, Middle SDI regions showed a slight increase with an EAPC of 0.01 per 100,000 population (95% CI: −0.03 to 0.05), which was not statistically significant. Conversely, regions with a high SDI had an EAPC of −0.03 per 100,000 population (95% CI: −0.07 to 0.01). Regions with a high-middle SDI had an EAPC of −0.09 per 100,000 population (95% CI: −0.14 to −0.04). Regions with a low-middle SDI had an EAPC of −0.07 per 100,000 population (95% CI: −0.1 to −0.05). SDI regions exhibited an EAPC of −0.07 per 100,000 population (95% CI: −0.1 to −0.05), while low SDI regions demonstrated an EAPC of −0.07 per 100,000 population (95% CI: −0.09 to −0.05), indicating a declining trend (Table 1).

Analysis of the 30-year prevalence trends across various continents revealed diverse patterns In Africa and America, the AAPCs were above zero, indicating an increasing prevalence of CKD. Conversely, in Asia, Europe, and Oceania, the AAPCs were below zero, indicating a general decline in CKD prevalence (Table 6).

In 2021, the ASIR of CKD exhibited an upward trajectory across diverse geographical regions and SDI areas, as illustrated in Table 2. Globally, Central Latin America exhibited the highest ASIR at 411.41 per 100,000 population (95% UI: 390.17–431.32), closely followed by North Africa and the Middle East at 411.19 per 100,000 population (95% UI: 385.4–438.45) (Figure 2B). From 1990 to 2021, the most significant increase in ASIR occurred in Andean Latin America, with an EAPC of 2.27 per 100,000 population (95% CI: 2.17–2.38). Consequently, among the various SDI regions, high SDI regions exhibited the highest ASIR in 2021, at 277.75 per 100,000 population (95% UI: 260.7–295.01), whereas Middle SDI regions demonstrated the most pronounced increase in ASIR from 1990 to 2021, with an EAPC of 1.06 per 100,000 population (95% CI: 1.04–1.08) (Table 2). Concurrently, the overall incidence trends across continents demonstrated consistent AAPCs exceeding zero (Table 6).

In 2021, the ASMR of CKD was highest in Central Sub-Saharan Africa at 43.69 per 100,000 population (95% UI: 33.26–56.29), followed by Central Latin America at 42.35 per 100,000 population (95% UI: 38.32–47.03), and Eastern Sub-Saharan Africa at 40.09 per 100,000 population (95% UI: 35.57–46.03) (Figure 2C). Similarly, the ASMR was highest in low SDI regions, at 29.43 per 100,000 population (95% UI: 26.13–33.79), followed by low-middle SDI regions, at 23.08 per 100,000 population (95% UI: 20.97–26.31). Conversely, Eastern Europe exhibited the lowest ASMR at 5.22 per 100,000 population (95% UI: 4.73–5.82). The ASMR was lower in both the High-middle SDI and High SDI regions, at 12.02 per 100,000 population (95% UI: 10.68–13.38) and 14.11 per 100,000 population (95% UI: 12.3–15.21), respectively (Table 3). It is noteworthy that, between 1990 and 2021, the High-income North America region exhibited the most pronounced increase in ASMR, with an EAPC of 3.4 per 100,000 population (95% CI: 3.2–3.6). In contrast, East Asia displayed the most notable decrease, with an EAPC of −0.84 per 100,000 population (95% CI: −0.92 to −0.76). Conversely, regions with a high SDI exhibited a significant increase in ASMR, with an EAPC of 1.73 per 100,000 population (95% CI: 1.61–1.86). In contrast, regions with a low SDI were the only SDI area to show a decrease, with an EAPC of −0.08 per 100,000 population (95% CI: −0.2 to 0.03) (Table 3). Furthermore, the overall mortality trends across continents demonstrated an increase with AAPCs greater than 0. Nevertheless, this uptrend was not statistically significant in Asia (AAPC = 7, 95% CI: −5.1 to 19.1%, *p* = 0.259) (Table 6).

The ASDR of CKD in 2021 was highest in Central Latin America, with a rate of 1,171.14 per 100,000 population (95% UI: 1,054.82–1,316.26). This was followed by Central Sub-Saharan Africa, with a rate of 1,124.7 per 100,000 population (95% UI: 899.03–1,436.09) (Table 4; Figure 2D). It is noteworthy that the two regions exhibited markedly disparate trends in change, with EAPCs of 1.75 per 100,000 population (95% CI: 1.32–2.18) and −0.19 per 100,000 population (95% CI: −0.26 to −0.12), respectively. The ASDR trends across regions categorized by SDI revealed patterns correlated with geographical patterns. Low SDI regions recorded the highest rates in 2021 at 791.8 per 100,000 population (95% UI: 704.14–909.1), with a declining EAPC of −0.34 per 100,000 population (95% CI: −0.42 to −0.25). By contrast, the High-middle SDI regions exhibited the lowest rate, at 324.64 per 100,000 population (95% CI: 293.58–360.92), with a similar downward EAPC trend of −0.35 per 100,000 population (95% CI: −0.42 to −0.28). In contrast, the other three SDI regions demonstrated increasing trends in ASDR (Table 4). Conversely, the overall DALYs trend in Asia diverged from the global and other continental trends, exhibiting an AAPC less than zero, indicative of a reduction in the burden of CKD-related health over the past three decades. This contrasts with global and other continental trends, suggesting that significant progress has been made in managing and treating CKD in the Asia region, with a reduction in associated health burdens (Table 6).

### Heterogeneity of CKD types

This study outlines the proportions of CKD and its subtypes caused by specific etiologies from a global perspective before delving into regional trends from 1990 to 2021.

This analysis primarily focuses on ASPR as the central metric, with additional indicators such as ASIR, ASMR, and ASMR included to provide supplementary insights into the global and regional CKD burden.

**Figure 3:**
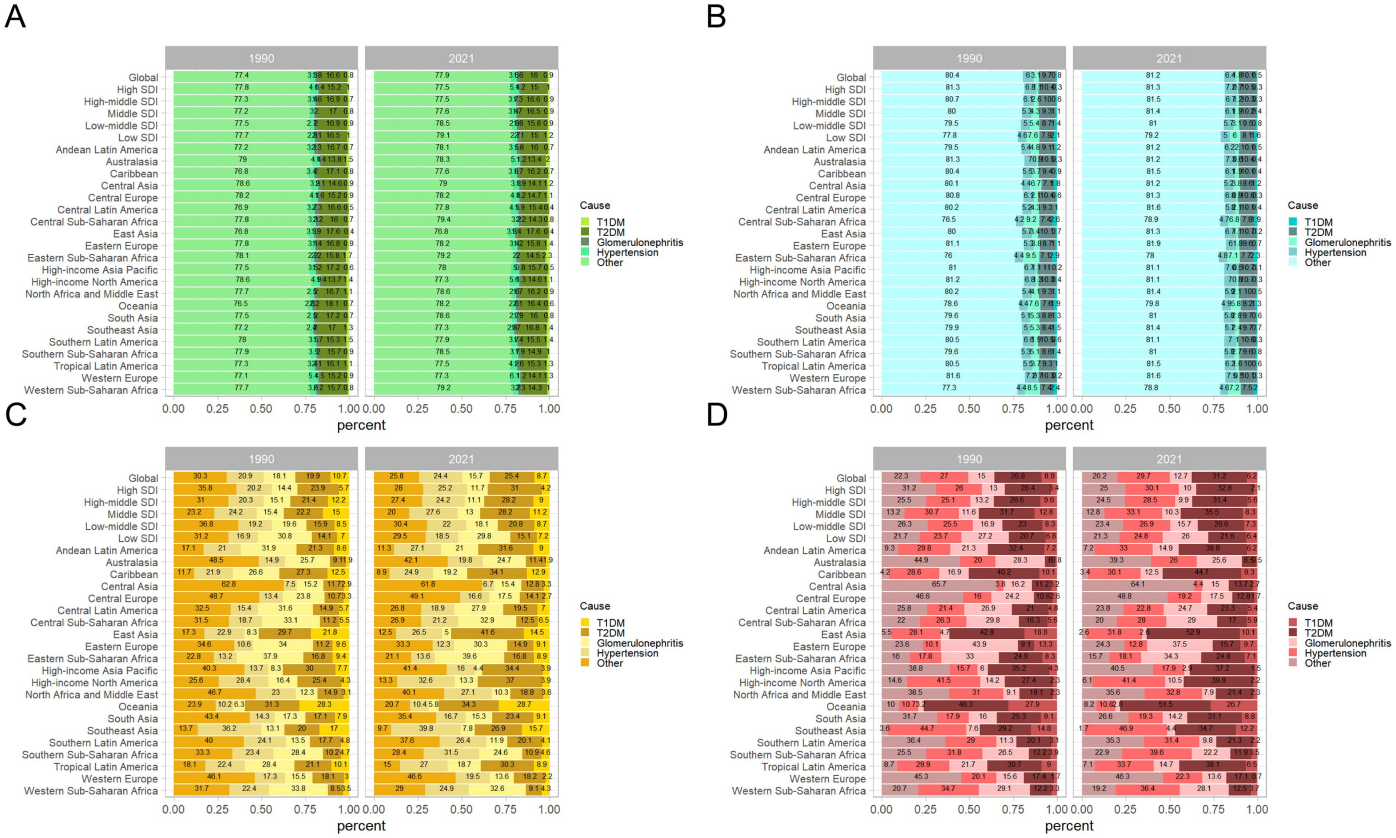
Contribution of T1DM, T2DM, glomerulonephritis, hypertension, and other causes to the disease burden of CKD, both sexes, globally and by region, in 1990 and 2021. **(A)** Contribution to prevalence cases of CKD; **(B)** Contribution to incidence cases of CKD; **(C)** Contribution to mortality cases of CKD; **(D)** Contribution to DALYs cases of CKD. CKD, chronic kidney disease; DALYs, disability-adjusted life years; T1DM, type 1 diabetes mellitus; T2DM, type 2 diabetes mellitus.

Globally, based on the ASPR, it is evident that over 70% of CKD cases in 1990 and 2021 were due to unspecified causes, followed by CKD induced by T2DM, CKD due to hypertension, CKD related to glomerulonephritis, and CKD caused by T1DM (Figure 3A). Among them, CKD stemming from unspecified causes remained the most dominant subtype, with an incidence rate of 6,247.94 per 100,000 (95% UI: 5,823.88–6,691.38) in 2021. CKD caused by T2DM was the second most prevalent subtype, with an incidence rate of 1,259.63 per 100,000 (95% UI: 1,161.99–1,359.92) in 2021. These two subtypes collectively account for the majority of new incidence rates in 2021 (Table 5; Figure 3A). Over time, the proportions of the different subtypes of CKD have remained relatively stable globally, although significant regional variations have occurred (Figure 3).

Regionally, there are notable differences in CKD subtype trends. For instance, in Australasia, the proportion of CKD caused by T2DM decreased from 13.8% in 1990 to 13.4% in 2021, while CKD caused by T1DM increased from 1.5 to 2% during the same period. In low-middle SDI regions, the proportion of CKD caused by other factors increased from 77.5 to 78.5%, while the proportion of CKD caused by T2DM decreased from 16.9 to 15.8% (Figure 3A).

**Figure 4:**
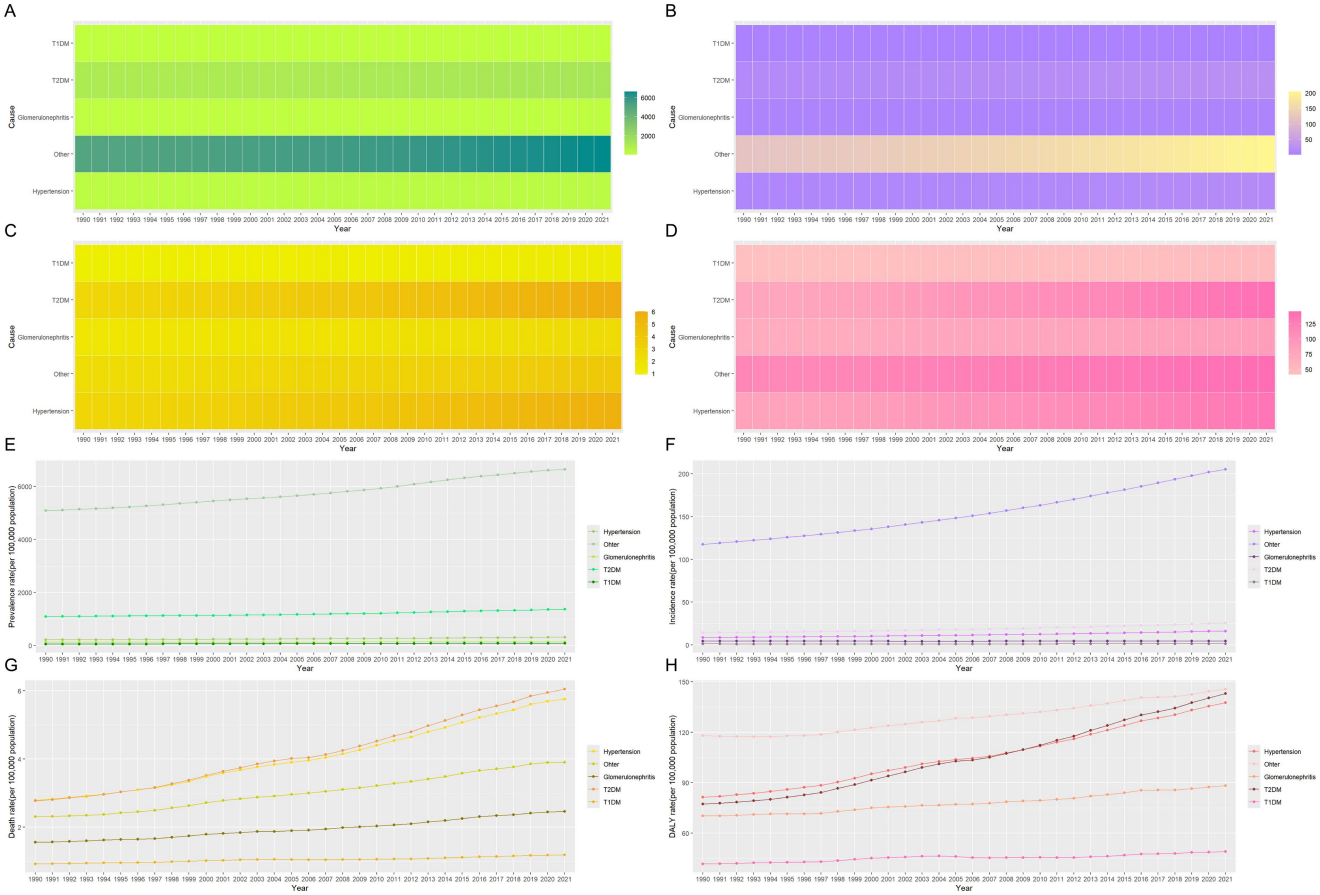
Heat map and development trends for the five subtypes disease indicators of CKD from 1990 to 2021. **(A)** The prevalence of five subtypes of CKD; **(B)** The incidence of five subtypes of CKD; **(C)** The mortality of five subtypes of CKD; **(D)** The DALYs of five subtypes of CKD; **(E)** Changes in the prevalence of five subtypes of CKD; **(F)** Changes in the incidence of five subtypes of CKD; **(G)** Changes in the mortality of five subtypes of CKD; **(H)** Changes in the DALYs of five subtypes of CKD. CKD, chronic kidney disease; DALYs, disability-adjusted life years; T1DM, type 1 diabetes mellitus; T2DM, type 2 diabetes mellitus.

A long-term trend analysis of global CKD subtypes indicates that the rising incidence rates are primarily driven by increased detection of CKD due to glomerulonephritis (EAPC = 0.39 per 100,000, 95% CI: 0.36–0.43) and CKD from unspecified causes (EAPC = 0.04 per 100,000, 95% CI: 0.01–0.07) (Table 5; Figures 4A,E). Consequently, the incidence of CKD subtypes exhibiting rising trends includes that of CKD caused by T1DM (AAPC = 95.6, 95% CI: 84.7–106.5%, *p* < 0.001), CKD caused by glomerulonephritis (AAPC = 4.1, 95% CI: 2.7–5.4%, *p* < 0.001), and CKD caused by other factors (AAPC = 0.7, 95% CI: −1.6 to 3.1%, *p* = 0.538) (Table 6). This may be indicative of heightened difficulties in the global management of diabetes and enhanced diagnostic capabilities for glomerulonephritis during this period.

Similarly, on a global scale, the top three CKD types with the highest ASIR in 2021 are CKD caused by unspecified factors (189.36 per 100,000), glomerulonephritis (129.94 per 100,000), and T1DM (77.31 per 100,000), as shown in Table 5. Figure 3B further illustrates the contribution of various causes to the CKD incidence rate burden. The significant increase in CKD incidence is primarily attributed to improved detection rates, particularly in cases linked to hypertension, unspecified causes, and T2DM, with respective rates of 0.66 per 100,000, 0.65 per 100,000, and 0.61 per 100,000 (Table 5; Figures 4B,F). Additionally, global long-term trend analysis shows significant increases in CKD cases caused by hypertension (AAPC = 65.4, 95% CI: 63.8–66.9%, *p* < 0.001), unspecified causes (AAPC = 64.1, 95% CI: 62.8–65.4%, *p* < 0.001), and T2DM (AAPC = 61.7, 95% CI: 59.8–63.7%, *p* < 0.001) (Table 6), underscoring that hypertension, unspecified causes, and T2DM are the primary factors driving the rise in CKD incidence rates.

Furthermore, based on the ASMR, it can be observed globally that by 2021, the proportion of CKD caused by T2DM has increased from 26.8 to 31.2%, while CKD caused by hypertension has increased from 27 to 29.7%. In contrast, other CKD subtypes have shown decreases (Figure 3C). In regions such as East Asia, High-income North America, and Southeast Asia, the combined proportion of CKD caused by T2DM and hypertension has escalated to as much as 80% (Figure 3C). These two types of CKD are the most prevalent contributors to mortality rates, with CKD caused by T2DM having a rate of 5.72 per 100,000 (95% UI: 4.83–6.79) and CKD caused by hypertension with a rate of 5.54 per 100,000 (95% UI: 4.68–6.41) (Table 5; Figure 4C). Additionally, significant changes in mortality trends for different CKD subtypes from 1990 to 2021 are highlighted in Figures 4C,G. A long-term global mortality trend analysis reveals a particularly significant increase in mortality rates associated with CKD caused by T2DM (AAPC = 106.9, 95% CI = 92.8–120.9%, *p* < 0.001), positioning it as one of the primary driving factors for the increasing global mortality related to CKD (Table 6).

Furthermore, based on ASDR, it was observed globally in 2021 that CKD caused by T2DM and hypertension each accounted for approximately one-fourth of the total CKD cases, comparable to CKD caused by other factors (Figure 3D). The rise in DALY rates is primarily due to enhanced detection of CKD caused by T2DM and hypertension, with respective rates of 0.8 per 100,000 and 0.62 per 100,000 (Figure 4D). Notably, from 1990 to 2021, only CKD caused by T1DM showed a significant decline in ASDR, with an EAPC of −0.21 per 100,000 (Table 5; Figures 4D,H). The global analysis reveals that while DALY rates for CKD and other subtypes have increased, T1DM and other causes have seen declines. The observed decrease in CKD incidence among individuals with T1DM suggests improvements in its management and treatment, warranting further research to identify strategies for enhancing prevention, treatment, and management across CKD types to reduce health burdens and disabilities.

### Gender and age structure and temporal trends

**Figure 5:**
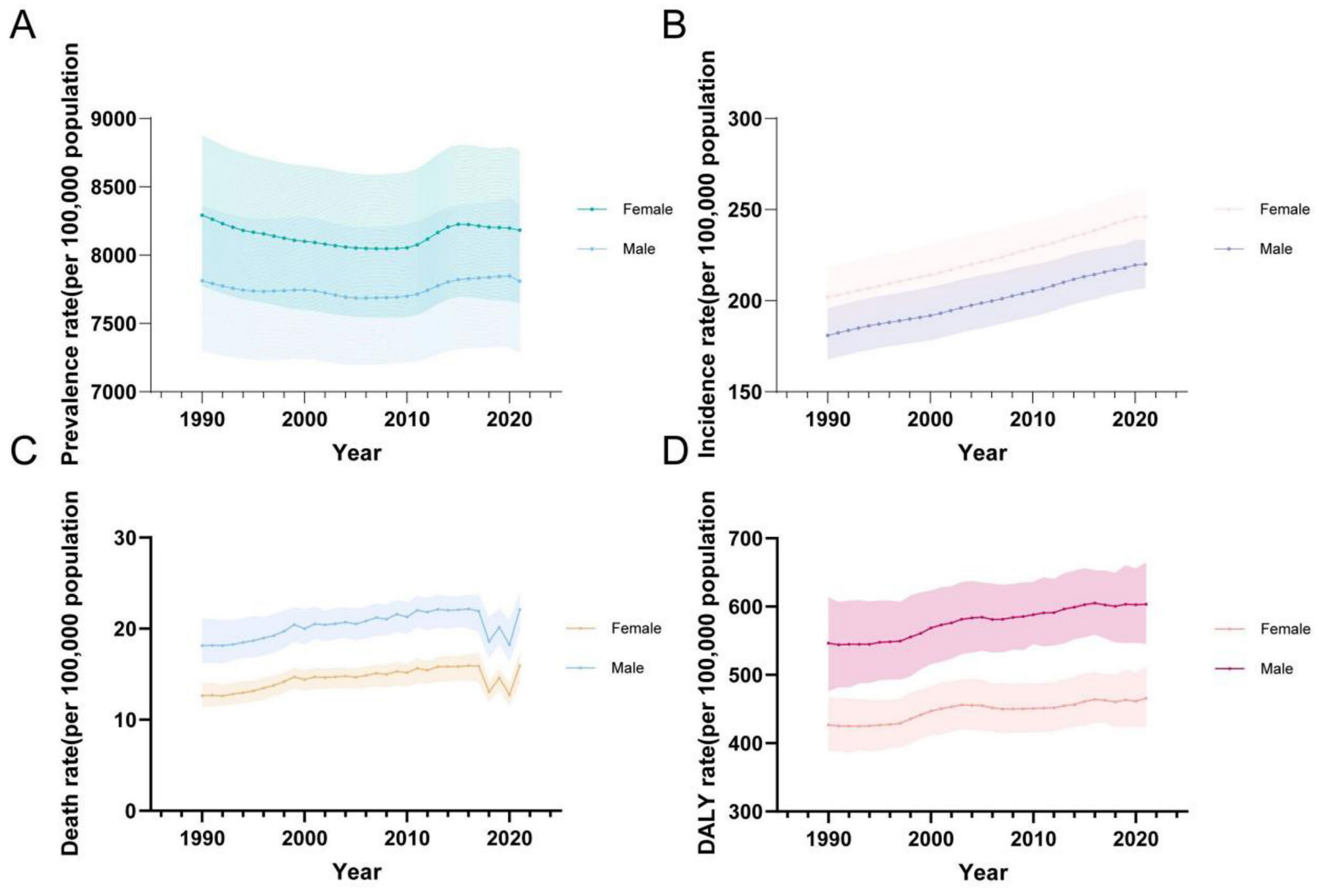
Global temporal trends in CKD disease burden, 1990–2021. **(A)** ASPR in different sexes; **(B)** ASIR in different sexes; **(C)** ASMR in different sexes; **(D)** ASDR in different sexes. CKD, chronic kidney disease; DALY, disability-adjusted life year; ASPR, Age standardized prevalence rate; ASIR, Age standardized incidence rate; ASMR, Age standardized mobility rate; ASDR, Age standardized DALYS rate.

For both sexes and all age groups overall, from 1990 to 2021, the ASPR of CKD demonstrated a general decline, while the ASIR, ASMR, and ASDR exhibited a general upward trend (Figure 5). It is notable that the ASPR initially followed a downward trend, with a slight increase around 2010 before decreasing again (Figure 5A). In contrast, ASIR showed a steady and continuous increase (Figure 5B). ASMR initially exhibited fluctuating increases, followed by a significant decline and rebound observed from 2017 to 2021, resulting in a noticeable fluctuating increase (Figure 5C). Similarly, ASDR rates exhibited a fluctuating upward trend (Figure 5D).

In terms of gender, it is noteworthy that, between 1990 and 2021, females consistently had higher ASPR and ASIR rates compared to males, whereas males exhibited higher ASMR and ASDR rates (Table 5; Figure 5).

**Figure 6:**
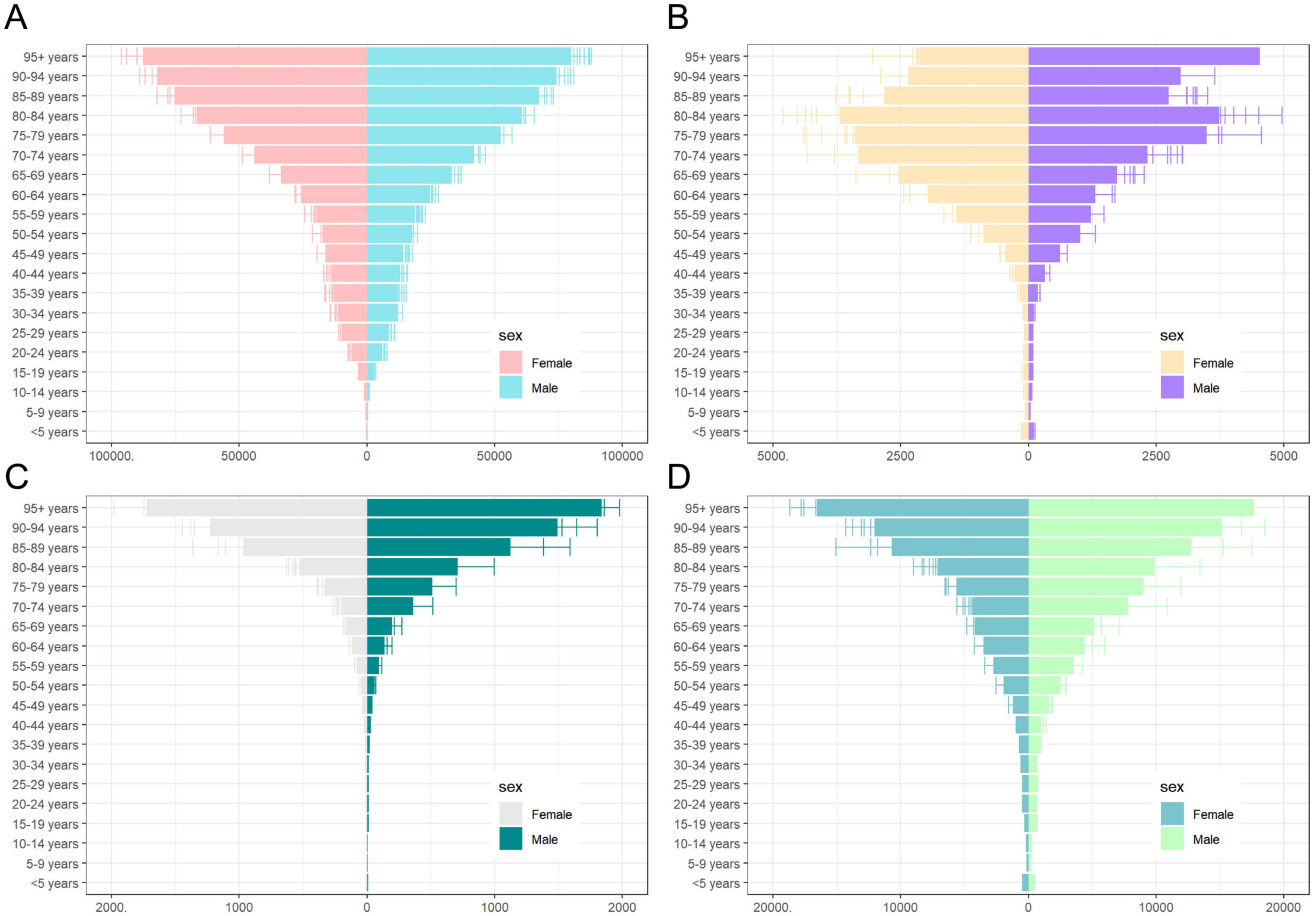
Sex and age-structured analysis of CKD disease burden in 2021. **(A)** Prevalence rates; **(B)** Incidence rates; **(C)** Mortality rates; **(D)** DALYs rates. CKD, chronic kidney disease; DALYs, disability-adjusted life years.

Regarding ASPR, females had a higher rate of 8,182.65 per 100,000 (95% UI: 7,653.14–8,764.76), compared to males at 7,808.96 per 100,000 (95% UI: 7,288.71–8,366.6) (Table 1). Prevalence rates were generally higher in females across most age groups, except for the 10–24 and 90+ age groups, where males showed higher rates (Figures 5A, 6A). The greatest disparity in prevalence rates between the sexes was observed in the 15–19 age group, while the smallest difference was seen in the 20–24 age group.

A similar pattern was observed for ASIR, with females at 246.03 per 100,000 (95% UI: 231.84–260.67), compared to males at 220.11 per 100,000 (95% UI: 207.07–233.07) (Table 2). No significant gender difference was observed across age groups for ASIR (Figure 6B).

In terms of ASMR, males had consistently higher mortality rates (21.91 per 100,000, 95% UI: 19.66–23.60) than females (16.9 per 100,000, 95% UI: 14.22–17.27) (Table 3). The largest gender difference in mortality rates was observed in the 5–9 age group, while the smallest difference occurred in the 10–14 age group (Figure 6C).

Finally, ASDR rates were higher in males (603.4 per 100,000, 95% UI: 546.08–663.35) compared to females (465.69 per 100,000, 95% UI: 424.02–511.12) (Table 4). The DALY trend mirrored that of mortality, with males consistently exhibiting higher rates across age groups. The greatest disparity in DALYs between the sexes was seen in the 5–9 age group, while the smallest difference was in the 10–14 age group (Figure 6D).

Reflecting on age groupings, in 2021, all CKD burden indicators showed an increase with age, with sharp rises observed beyond 65 years old. The group aged 95 and older had the highest rates for prevalence, mortality, and DALYs, while the 80–84 age group reported the highest incidence rates (Figure 6).

### Temporal joinpoint analysis

**Figure 7:**
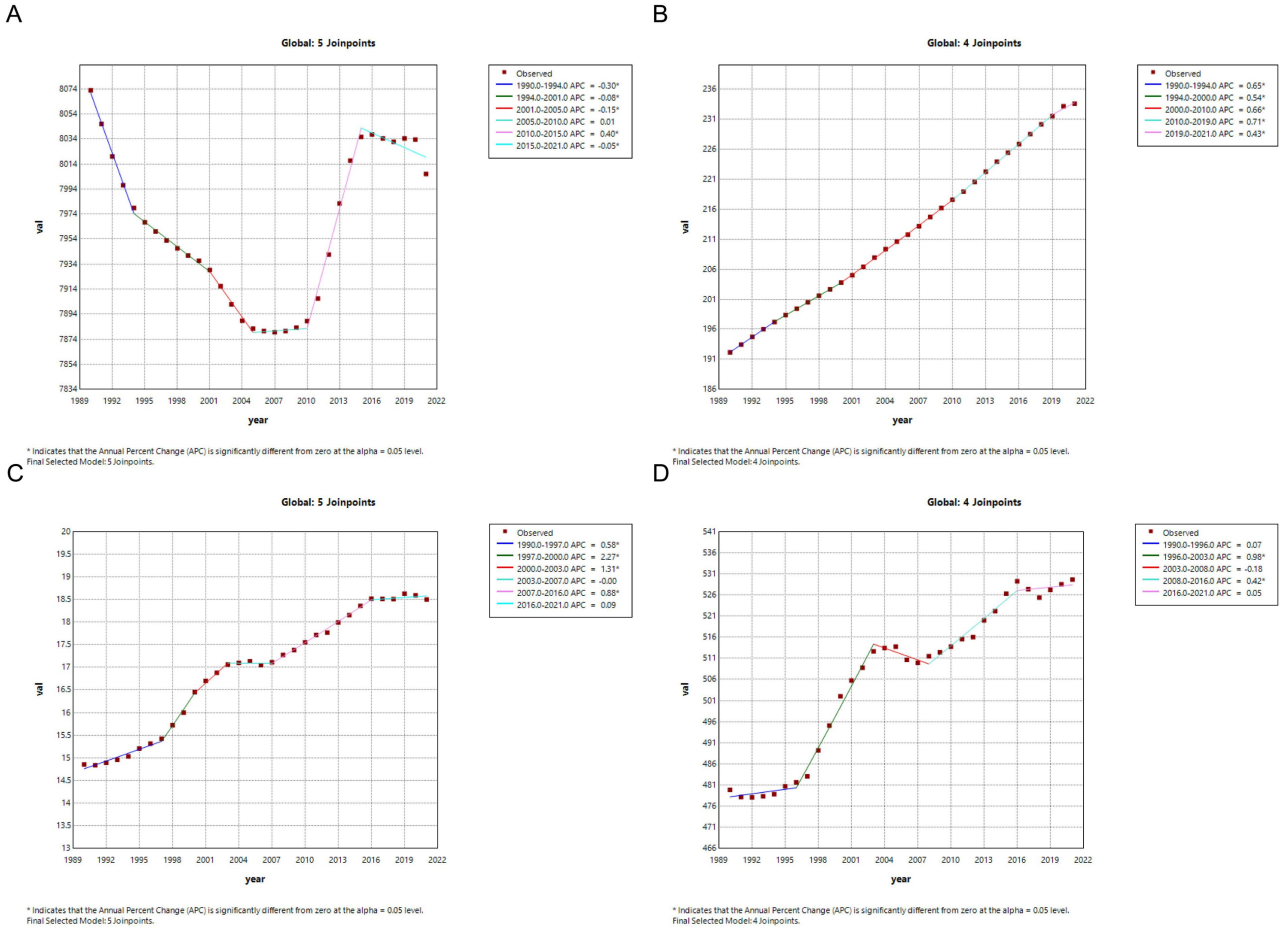
Joinpoint regression analysis of the CKD disease burden temporal trends, 1990–2021. **(A)** ASPR; **(B)** ASIR; **(C)** ASMR; **(D)** ASDR. CKD, chronic kidney disease; DALYs, disability-adjusted life years; ASPR, Age standardized prevalence rate; ASIR, Age standardized incidence rate; ASMR, Age standardized mobility rate; ASDR, Age standardized DALYS rate.

The results of the joinpoint regression analysis indicate that the ASPR of CKD globally exhibited a downward trend over the period from 1990 to 2021. However, an upward trend was observed during the period from 2010 to 2015 (APC = 40.3, 95% CI: 34.7–45.9%, *p* < 0.001) (Table 6; Figure 7A). In contrast, the ASIR demonstrated a significant and steady increase globally over the 30-years period, with the most notable increase observed between 2010 and 2019 (APC = 70.6, 95% CI: 69.1–72%, *p* < 0.001) (Table 6; Figure 7B).

Similarly, the ASMR demonstrated a significant global increase over the 30-year period, with a notably steep rise during 1997–2000 (APC = 226.9, 95% CI: 128–326.7%, *p* < 0.001). However, a slight decrease was observed from 2003 to 2007, which was not statistically significant (APC = −0.5, 95% CI: −45.3 to 44.5%, *p* = 0.982) (Table 6; Figure 7C). The trends in ASDR mirrored those of ASMR, showing a similar upward trend over 30 years, with a substantial increase during 1996–2003 (APC = 98.2, 95% CI: 85.6–110.7%, *p* < 0.001). However, a slight non-significant decrease was observed from 2003 to 2008 (APC = −18.4, 95% CI: −41 to 4.3%, *p* = 0.106) (Table 6; Figure 7D).

In different regions, the overall trends of CKD burden indicators are generally aligned with global patterns. However, some regions exhibit phenomena that are in opposition to these global trends. For example, between 1990 and 2021, the ASPR increased in Africa (AAPC = 3.2, 95% CI: 2.7–3.7%, *p* < 0.001), America (AAPC = 4.5, 95% CI: 0.9–8.1%, *p* = 0.015), and Oceania (AAPC = 8.7, 95% CI: 7.7–9.6%, *p* < 0.001), contrasting with the global trend. Similarly, the ASDR exhibited a decline in Asia (AAPC = −19, 95% CI: −27.2% to −10.8%, *p* < 0.001), which contrasts with the global increase (Table 6).

As expected, there are discrepancies in the prevalence of CKD indicators across regions over the same temporal period. For example, with regard to ASPR, there was a decrease in Asia (APC = −26, 95% CI: −31.3% to −20.7%, *p* < 0.001) from 1994 to 2000, while America experienced an increase (APC = 22.3, 95% CI: 14.6–30%, *p* < 0.001) during the same period (Supplementary Figure 1).

From 1990 to 2021, a comparison of five different subtypes of CKD worldwide revealed significant differences in disease burden indicators in terms of prevalence and DALYs compared to the overall CKD population. For instance, the global AAPC in the prevalence of CKD caused by T1DM (AAPC = 95.6, 95% CI: 84.7–106.5%, *p* < 0.001), CKD caused by glomerulonephritis (AAPC = 4.1, 95% CI: 2.7–5.4%, *p* < 0.001), and CKD caused by other (AAPC = 0.7, 95% CI: −1.6 to 3.1%, *p* = 0.538) showed an upward trend, which contrasts with the overall decline in CKD prevalence. Similarly, the global AAPC in DALYs for CKD caused by T1DM (AAPC = −11.9, 95% CI: −20.9% to −3%, *p* = 0.009) and CKD caused by other (AAPC = −1.6, 95% CI: −8.8 to 5.6%, *p* = 0.655) showed a decrease, whereas the overall CKD DALYs increased (Table 6).

Nevertheless, other indicators, such as incidence and mortality rates, did not show significant global changes, particularly between subtypes, where differences were not pronounced. Therefore, the research perspective shifts from a global to a regional analysis, with a particular focus on incidence and mortality rates.

The study reveals notable regional differences in the prevalence of CKD subtypes compared to global trends. For instance, in Europe, the incidence of CKD caused by glomerulonephritis is significantly lower than that of other causes, which are closer to global levels (Supplementary Figure 2). This suggests that regional factors, such as healthcare access, diagnostic capabilities, and genetic predispositions, may play a significant role in shaping the prevalence of CKD subtypes in Europe, contrasting with global trends. Regarding mortality rates, while all CKD subtypes showed an increasing trend globally, in both Africa and Europe, CKD caused by T1DM demonstrated a decreasing trend, which is in contrast to the rising mortality trends observed in the other subtypes (Supplementary Figures 3, 4). This divergence is an interesting phenomenon, highlighting the distinct mortality trends of T1DM-induced CKD compared to other subtypes. It may reflect regional differences in healthcare interventions or lifestyle factors that specifically influence T1DM-related CKD mortality.

Additionally, the study further conducted a year-by-year analysis over the past 30 years and found that, despite an overall trend across the three decades, specific years within this period showed deviations from the general trend. This suggests that factors such as healthcare advancements, changes in medical policies, improvements in diagnostic capabilities, and even socio-economic conditions may have influenced the trends on a more localized or short-term scale. For instance, differences in the annual percentage change in ASPR for CKD caused by T1DM during 2015–2021 revealed contrasting trends across regions: Europe saw a dramatic increase (APC = 146, 95% CI: 139.1–153%, *p* < 0.001), while America experienced a significant decrease (APC = −46.3, 95% CI: −55.4% to −37.3%, *p* < 0.001) (Supplementary Figure 5). These regional variations highlight how healthcare factors and public health interventions can lead to different trends in CKD prevalence and mortality across different parts of the world, even when the overall global trend suggests a more consistent pattern. Such anomalies underscore the need to consider both long-term global trends and regional-specific factors when analyzing disease burdens.

## Discussion

CKD is a significant global public health concern (11, 12), with increasing incidence rates and mortality, as well as a contributory factor in the development of systemic diseases such as ocular (13), neurological (14), and cardiovascular diseases (15–17). Despite substantial research on CKD (18–20), comprehensive literature analyses covering the global burden, including prevalence, incidence, mortality, and DALYs for CKD and its subtypes, are lacking. Most previous studies have focused on specific age groups, subtypes, or countries (2, 9, 21). This study aims to fill this gap by providing a broad, up-to-date analysis spanning 32 years, covering 204 countries and territories across all age groups, sexes, and SDI levels. The findings underscore the importance of continued global efforts in managing CKD and its subtypes, highlighting the need for updated data to inform policymaking and effective interventions.

### Global trends in CKD epidemiology

In 2021, global CKD incidence reached 11,128,691 cases, with prevalence at 358,777,424 cases. Mortality attributed to CKD was 1,527,639 deaths, and DALYs totaled 44,453,684. Compared to 1990, these figures represent significant increases. However, due to the considerable uncertainty surrounding these figures, caution is warranted in interpreting them. Joinpoint regression analysis reveals that while overall prevalence has decreased slightly, incidence, mortality, and DALYs rates continue to rise, reflecting increasing CKD burden. Despite this, the decrease in prevalence over time may reflect improvements in healthcare, preventive measures, and treatment options globally, which have led to more effective management, even as the overall disease burden escalates.

### Population dynamics and key drivers of the global CKD burden

The global burden of CKD is driven by more than just population growth; various demographic and epidemiological factors also play a crucial role. A 47% increase in global population, according to the 2022 Revision of World Population Prospects, contributes significantly to the rise in CKD cases, despite stable prevalence and incidence rates. Changes in disease incidence, survival rates, and life expectancy also influence CKD prevalence, with mortality and DALY rates closely tied to these factors (22).

The increase in CKD prevalence is particularly marked in middle SDI regions, which are more vulnerable to CKD’s impact (22). This trend is driven by demographic factors such as population growth, migration, and birth rates. Previous studies highlight the role of treatment modalities (23), dietary habits (24), nutritional management (25), healthcare access (26), and lifestyle behaviors (27) in shaping CKD burdens. These findings underscore the importance of equitable resource distribution to mitigate CKD’s impact, especially in lower-income countries. Additionally, joinpoint regression analysis highlights geographical disparities in CKD trends, suggesting the need for region-specific public health interventions.

However, the rising CKD prevalence is not solely attributable to population growth. Aging populations, urbanization, and the increasing prevalence of risk factors such as diabetes and hypertension also contribute significantly. These demographic shifts, along with environmental and lifestyle factors and advances in healthcare, improve CKD detection. While population growth is responsible for the increase in absolute CKD numbers, further research is needed to better understand the roles of aging, chronic diseases, and other factors in shaping the global CKD burden.

### Major CKD risk factors and public health challenges

The growing global burden of CKD is exacerbated by major risk factors such as diabetes, hypertension, and glomerulonephritis (28, 29). The rapid rise in diabetes cases, as indicated by the 2022 International Diabetes Federation Diabetes Atlas,^3^ contributes significantly to CKD, with projections suggesting 783 million diabetes patients by 2045 (30). Both Type 1 and Type 2 diabetes are crucial contributors to CKD, with diabetes-related complications requiring continuous management, adding to the strain on healthcare systems (31, 32). The prevalence and incidence of CKD caused by T1DM have notably increased over the past three decades, underscoring its emerging significance as a public health issue. Early detection and intervention are essential for preventing CKD in T1DM patients (2, 22). On the other hand, T2DM remains a major CKD risk factor (33, 34), with substantial global research investigating the T2DM-CKD relationship (35–39).

Hypertension, another well-established CKD risk factor, is linked to CKD progression, though the role of primary hypertension in end-stage renal disease (ESRD) remains under debate (40). Elevated blood pressure may point to underlying genetic factors or pathophysiological conditions, such as APOL1 nephropathy (41–43), vascular calcification (44), or reduced renal unit endowment (45), contributing to CKD and increasing its burden, especially in regions with limited healthcare resources (46, 47).

Although less common, glomerulonephritis is a lifelong cause of CKD (48), with a significant impact on patient quality of life (49). Studies have shown that the complex interaction of lncRNA and epigenetic modifications in glomerulonephritis complicates its management (50). Patients affected by CKD caused by glomerulonephritis experience severe impacts on emotional, social, physical, and economic aspects (51), and it is particularly prevalent in middle-to-low-income countries, contributing to global CKD disparities (52). Additionally, CKD cases from other undiagnosed causes present unique challenges due to the lack of standardized treatment protocols, highlighting the need for coordinated global healthcare policies to improve kidney disease care (53, 54).

### Regional disparities and trends

CKD trends show significant variation across regions, influenced by factors such as SDI levels, healthcare access, and demographic transitions (55, 56). The Middle SDI region, for example, has seen a steady increase in CKD prevalence, driven by factors like aging populations, migration, dietary shifts, and economic development (57). This region, positioned between low and high SDI levels, faces unique challenges related to healthcare infrastructure and disease patterns, such as the shift from infectious to chronic diseases.

In contrast, high-middle and high SDI regions, while experiencing an aging population and rising chronic disease burden, benefit from better disease detection and healthcare systems. These improvements have led to more accurate CKD diagnosis and management, resulting in slower increases in CKD prevalence despite the growing number of cases. Some regions, like parts of Asia and Europe, have even seen a decline in CKD prevalence, reflecting the success of public health measures (58–60).

However, disparities persist within regions. In the Americas, socioeconomic factors, unequal healthcare coverage, and regional health challenges contribute to uneven CKD outcomes, particularly among disadvantaged populations (57–59). These variations underscore the need for region-specific CKD strategies that consider local economic, demographic, and healthcare conditions to improve global and regional health outcomes.

### Age and gender disparities

Pediatric CKD, although sharing similarities with adult CKD, presents unique challenges due to its impact on children’s growth, development, and long-term health. Boys are generally more affected by CKD than girls, with higher mortality and DALY rates observed in male children (61) Globally, the incidence of CKD in children ranges from 15 to 74.7 cases per million, with the highest rates in middle- and low-income countries (62–64). Preventing and managing childhood CKD is crucial, as it affects not only renal health but also cardiovascular and metabolic functions (61, 65–67).

As individuals age, the burden of CKD increases. Middle-aged and elderly populations are at heightened risk due to the natural decline in kidney function and the cumulative impact of chronic diseases like hypertension and diabetes (68–74). For older adults, CKD not only impacts kidney health but also increases the risk of complications such as cardiovascular events, depression, and cognitive decline (22, 75–78),. Diagnosing CKD in this age group can be challenging, as they may not report symptoms as accurately as younger individuals, complicating early detection and management.

Gender-specific disparities in the burden of CKD exist not only in children but also in young adults and the elderly (79). Gender differences and stereotypes negatively impact various aspects of healthcare, leading to delays in diagnosis and treatment, decreased quality of care, and increased disease burden. Broadly speaking, these biases have a more severe impact on women than men, but any bias leads to health inequities and negatively affects everyone (80). Over three decades, ASPR and ASIR rates for CKD have consistently been higher in women than in men (81, 82). Studies suggest that as age increases, the natural decline in glomerular filtration rate due to extended life expectancy and potential overdiagnosis of CKD contribute to higher rates of CKD in women (83, 84). Women generally have a higher risk of incidence and prevalence of CKD over three decades, reflecting specific biological and social factors such as gender biases, hormone levels, reproductive history, and patterns of healthcare utilization (85). Additionally, studies indicate that women are less likely than men to recognize they have CKD (86, 87), factors that may increase the risk of CKD in women. A substantial body of research indicates that awareness of kidney disease is low not only in women but also in all populations (88). Therefore, there is an urgent need for carefully designed clinical trials to increase female representation, enhance knowledge of early detection of kidney disease, improve doctor-patient communication, update and use the CKD-EPI formula reasonably to ensure consistency and accuracy in global assessment of kidney function between genders (89), and prioritize gender and gender consideration factors in health decision-making (90). Conversely, ASMR and ASDR rates have consistently been higher in men than women (91, 92), possibly due to higher specific health risks such as cardiovascular disease and certain types of cancer, which are more common or severe in men. This is consistent with Vallianou et al.’s study, which found mutual risk factors between cardiovascular disease and chronic kidney disease include age, hypertension, diabetes, and male gender (93). Additionally, studies indicate that women have longer survival periods after kidney transplantation compared to men (79), which may also contribute to higher absolute cases of CKD-related deaths in men. In conclusion, these findings of gender disparities and stereotypes highlight the importance of considering gender factors in developing public health strategies and health interventions for CKD. Further research could explore biological and social differences between genders in CKD disease development and health outcomes to better tailor and optimize gender-specific healthcare measures.

## Limitations

It should be noted, however, that the study is not without limitations. Firstly, the study utilized the GBD 2021 database, which provides estimated data derived from a combination of system dynamics and statistical models rather than real observational data. This limitation implies that the estimates of disease burden may not be precise and may not reflect the most current data available. Secondly, GBD data are aggregated at a global and national level, lacking detailed data from specific regions, urban and rural areas, as well as specific demographic groups such as age, ethnicity, and socioeconomic status. This limitation could potentially affect the comprehensiveness and depth of the analysis of CKD disease burden. Finally, the study broadened the scope to include all types of CKD, yet it did not differentiate the specific burdens of each subtype nor further distinguish CKD caused by other conditions.

## Conclusion

In conclusion, this study employed data from the GBD 2021 to illustrate the global, regional, and national burden of CKD, analyzing trends from 1990 to 2021. The results indicate a slight decrease in the overall prevalence of CKD, but a further increase in the disease burden of the condition, which may be mainly caused by population growth and aging. The findings provide updated epidemiological information on CKD and emphasize the necessity for continual improvement in policies for diagnosing, treating, and managing CKD.

## Data Availability

The original contributions presented in the study are included in the article/Supplementary material, further inquiries can be directed to the corresponding authors.

## Footnotes

^1^Available at: https://ghdx.healthdata.org/gbd-2021/sources.

^2^Available at: https://www.healthdata.org/research-analysis/diseases-injuries-risks/factsheets/2021-chronic-kidney-disease-level-3-disease.

^3^Available at: http://www.diabetesatlas.org/.
