# Supplementary figures and images for: Global, regional, and national burden inequality of chronic kidney disease, 1990–2021: a systematic analysis for the global burden of disease study 2021
Source: Front Med (Lausanne). 2025 Jan 15;11:1501175. doi: 10.3389/fmed.2024.1501175 (PMC11774877; doi:10.3389/fmed.2024.1501175)

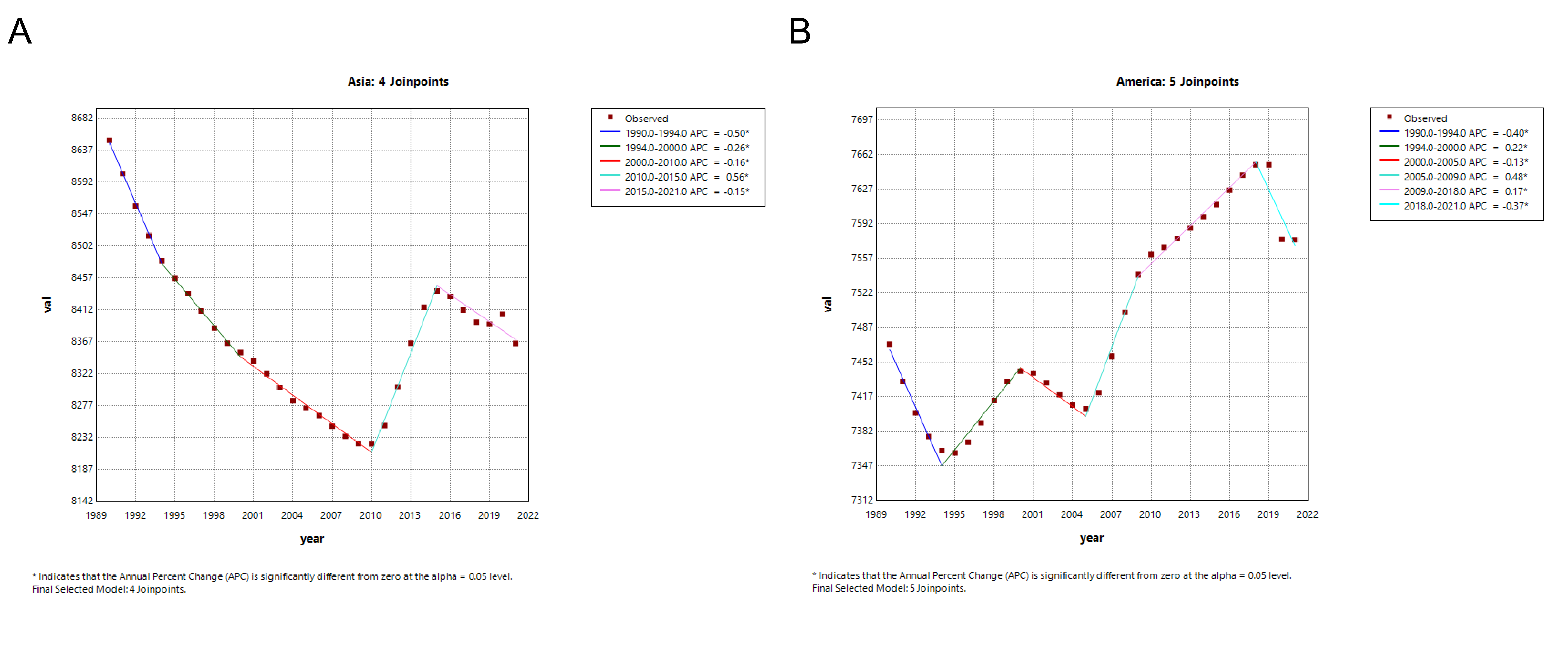

Supplement: Supplementary file 1 [file Image_1.TIF]

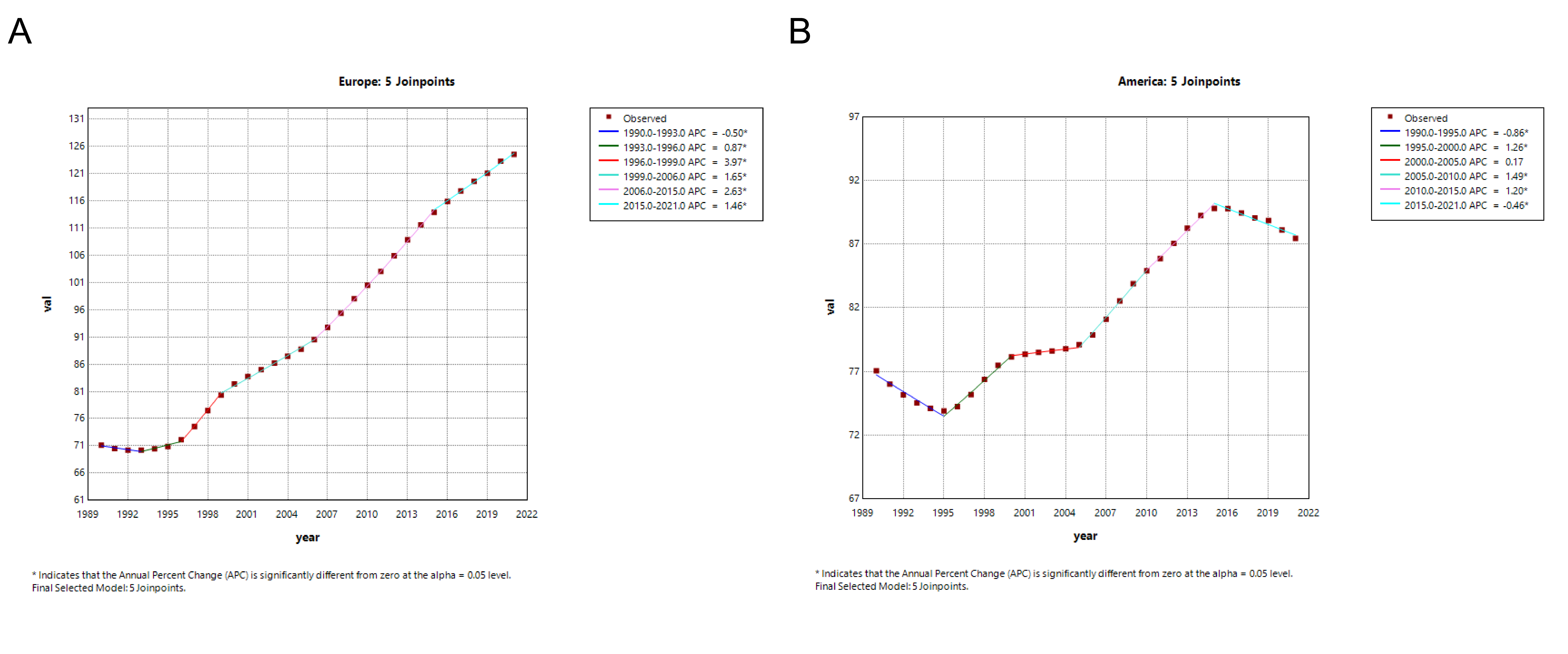

Supplement: Supplementary file 5 [file Image_5.TIF]
